# Multiple vector-borne pathogens of domestic animals in Egypt

**DOI:** 10.1371/journal.pntd.0009767

**Published:** 2021-09-29

**Authors:** Hend H. A. M. Abdullah, Nadia Amanzougaghene, Handi Dahmana, Meriem Louni, Didier Raoult, Oleg Mediannikov

**Affiliations:** 1 Department of Parasitology and Animal Diseases, Veterinary Research Division, National Research Centre, Dokki, Giza, Egypt; 2 Aix Marseille Univ, IRD, AP-HM, MEPHI, IHU-Méditerranée Infection, Marseille, France; Medical College of Wisconsin, UNITED STATES

## Abstract

Vector Borne Diseases (VBDs) are considered emerging and re-emerging diseases that represent a global burden. The aim of this study was to explore and characterize vector-borne pathogens in different domestic animal hosts in Egypt. A total of 557 blood samples were collected from different animals using a convenience sampling strategy (203 dogs, 149 camels, 88 cattle, 26 buffaloes, 58 sheep and 33 goats). All samples were tested for multiple pathogens using quantitative PCR and standard PCR coupled with sequencing. We identified *Theileria annulata* and *Babesia bigemina* in cattle (15.9 and 1.1%, respectively), *T*. *ovis* in sheep and buffaloes (8.6 and 7.7%, respectively) and *Ba*. *canis* in dogs (0.5%) as well as *Anaplasma marginale* in cattle, sheep and camels (20.4, 3.4 and 0.7%, respectively) and *Coxiella burnetii* in sheep and goats (1.7 and 3%; respectively). New genotypes of *An*. *centrale*, *An*. *ovis*, *An*. *platys*-like and *Borrelia theileri* were found in cattle (1.1,3.4, 3.4 and 3.4%, respectively), *An*. *platys*-like in buffaloes (7.7%), *An*. *marginale*, *An*. *ovis*, *An*. *platys*-like and *Bo*. *theileri* in sheep (3.4, 1.7, 1.7 and 3.4%, respectively), *An*. *platys*, *An*. *platys*-like and *Setaria digitata* in camels (0.7, 5.4 and 0.7%, respectively) and *Rickettsia africae*-like, *An*. *platys*, *Dirofilaria repens* and *Acanthocheilonema reconditum* in dogs (1.5, 3.4, 1 and 0.5%, respectively). Co-infections were found in cattle, sheep and dogs (5.7, 1.7, 0.5%, respectively). For the first time, we have demonstrated the presence of several vector-borne zoonoses in the blood of domestic animals in Egypt. Dogs and ruminants seem to play a significant role in the epidemiological cycle of VBDs.

## Author summary

Vector Borne Diseases (VBDs) are considered emerging and re-emerging diseases that represent a global burden. Diagnosis of these diseases is challenging due to nonspecific febrile illness, difficulty of isolation, and cross-reactivity of serological methods. Therefore, the current study is the first large-scale epidemiological study in which molecular screening and characterization of multiple vector-borne pathogens in different animal hosts were performed to better understand the endemicity of VBDs in Egypt. We detected for the first time *Anaplasma centrale*, *An*. *ovis*, a novel *An*. *platys*-like and *Borrelia theileri* in cattle, a new *An*. *platys*-like in buffaloes, *An*. *marginale*, *An*. *ovis*, a new *An*. *platys*-like and *Bo*. *theileri* in sheep, *An*. *platys*, a new *An*. *platys*-like and *Setaria digitata* in camels and *Rickettsia africae*-like, *An*. *platys*, *Dirofilaria repens* and *Acanthocheilonema reconditum* in dogs, in Egypt. These results imply that ruminants and dogs in Egypt are reservoirs for several neglected, emerging and re-emerging potentially new vector-borne pathogens that have significant implications in human health.

## Introduction

Vector Borne Diseases (VBDs) are emerging and re-emerging infectious diseases, that pose a health threat to humans, livestock, companion animals and wildlife [1]. VBDs are a global burden and cause severe economic losses through high mortality rates and production declines in the livestock industry, as well as impacts on human and animal health [2,3]. Moreover, about a quarter of vertebrate pathogens of veterinary importance are VBDs [4]. The World Organization for Animal Health (OIE) list includes many VBDs such as piroplasmoses, anaplasmoses and Q fever. The epidemiology and spread of VBDs are influenced by various factors such as globalization and increasing international trade, urbanization, climate change, travel and mobility of animals which pose unprecedented challenges to clinicians and veterinarians [5–6].

Piroplasmoses are tick-borne infectious diseases caused by apicomplexans of the order *Piroplasmida*, which includes three genera namely: *Theileria*, *Babesia* and *Cytauxzoon* [7]. *Theileria annulata*, *T*. *ovis* and *Babesia bigemina* are etiological agents of tropical theilerioses and babesiosis in ruminants especially cattle, buffalo and sheep [8]. Similarly, *Ba*. *canis* and *Ba*. *vogeli* are the main causative agents of canine babesiosis [9]. Piroplasmoses are common in Asia, Southern Europe and Africa [10]. The main clinical signs of piroplasmoses are fever and hemolytic anemia and deaths of up to 50% in the case of acute infection in susceptible herds [11,12]. Recovered animals may become asymptomatic carriers with long-term persistent infection [13,14]. Piroplasmoses have been detected in several provinces of Egypt and are widespread [15–18].

Anaplasmataceae include many tick-borne bacteria that infect mammals and consist of at least five genera: *Anaplasma*, *Ehrlichia*, *Neoehrlichia Neorickettsia*, and *Aegyptianella* [19–20]. Bovine anaplasmosis caused by *Anaplasma marginale* and *An*. *centrale* mainly in tropical and subtropical regions cause mild to severe anemia in ruminants [20,21]. Ovine anaplasmosis is a neglected mild disease in sheep, goats and wild ruminants caused by *An*. *ovis* and is common in different areas of the world [22,23]. In addition, there are many Anaplasmataceae bacteria pathogenic to dogs, such as *An*. *platys* and *Ehrlichia canis* [24,25]. Overall, these bacteria could cause persistent infection in mammals making them reservoir, which has lasting effect on the spread and new outbreaks of anaplasmosis [26,27]. In Egypt, anaplasmosis has been reported in cattle, water buffaloes and camels in different provinces [16,28–34].

Rickettsioses are bacterial infectious diseases that cause health problems in humans and animals worldwide [35,36]. Rickettsiae are divided into spotted fever group (SFG; mainly transmitted by ticks), typhus group (TG; transmitted by lice and fleas), *Rickettsia belli* group and *Rickettsia* (*R*.) *candensis* group [37]. *R*. *africae* is the most common rickettsial species in Africa that causes African tick-borne fever in humans [38]. Other rickettsiae such as *R*. *aeschlimannii*, *R*. *conorii and R*. *sibirica mongolitimonae*, *R*. *massiliae* have been detected in ticks and animals in Africa [39–43]. In Egypt, SFG have been identified in vectors, animals and humans since 1989 [44–48]. SFG rickettsiae were found in ticks (*Hyalomma* sp. and *Rhipicephalus sanguineus*) collected in Sinai province [49–51]. Moreover, *R*. *siberica mongolitimonae* was detected in a French traveler returning from Egypt [52]. Finally, *R*. *africae* was detected by molecular biology in *Hyalomma* sp. and camels [53–55].

Borrelioses are zoonotic infectious diseases and are divided into two groups: Lyme disease group (caused by *Borrelia burgdorferi* and related species) and relapsing fever group [56]. Relapsing fever borrelioses are arthropod-borne spirochetal diseases, usually transmitted by soft ticks; they are common in subtropical regions worldwide [57]. In Africa, relapsing fever is most common in the northern hemisphere and is caused by various *Borrelia* spp. such as *Bo*. *hispanica*, *Bo*. *duttonii*, and *Bo*. *crocidurae* [57–60]. *Bo*. *theileri* is the etiological agent of bovine borreliosis in ruminants, which causes anemia and fever and, unlike other members of the relapsing fever spirochetes, is transmitted by hard ticks [58]. In Egypt, data on borrelioses in animal hosts are sparse. Only the few studies have detected *Bo*. *burgdorferi* [61,62] and *Bo*. *theileri* in hard ticks [62].

Q fever is a zoonosis that infects humans and animals through direct contact or a tick bite [63]. *Coxiella burnetii* is the causative agent of Q fever that may be severe in humans [64]. Infection in animals it is usually subclinical except that reproductive diminution and abortions may occur [65]. *Coxiella burnetii* infects a wide range of animals, especially sheep, goats, cattle and camels, which serve as reservoirs [64,66]. In Egypt, the seroprevalence of *C*. *burnetii* was estimated in buffaloes, sheep, cattle and camels [67–70]. In addition, *C*. *burnetii* has been detected molecularly in goats, camels and ticks (*H*. *dromedarii*) [70–72].

Filarial nematodes are vector-borne helminths belonging to the order Spiruridae, suborder Spirurina and families Filariidae and Onchocercidae and pose a serious threat to humans and livestock [73,74]. *Dirofilaria repens* and *D*. *immitis*, followed by *Acanthocheilonema* sp. are the most important etiological agents of filarial infections in dogs [9,73,75]. *Setaria digitata* is a filarial nematode of cattle and buffaloes and is not pathogenic to these natural hosts, but when transmitted by mosquitoes to accidental hosts such as camels and horses, it can have serious pathological effects [76,77]. In Egypt, information on filarial infections in ruminants and dogs are scarce. In Africa, there are some reports of filarial infections in different places of the continent [78–80].

Diagnosis of all these diseases is challenging due to the non-specific febrile illness, difficulty in isolation and cross reactivity of serological methods [35,59]. Therefore, the advanced molecular techniques have been used to increase the sensitivity and specificity of diagnosis, to detect previously unknown pathogens and distinguish closely related species [5]. In Egypt, the epidemiology and prevalence of these diseases remain neglected and poorly understood. To date, few studies have been conducted on individual VBDs in vectors or animal hosts. Here, we provide the first data for molecular screening and characterization of multiple vector-borne pathogens in different animal hosts to better understand the epidemiological approach of VBDs in Egypt.

## Materials and methods

### Ethical approval

This study was approved by the Medical Research Ethics Committee at the National Research Centre, Egypt with the number 19058.

### Study area and samples collection

**Fig 1:**
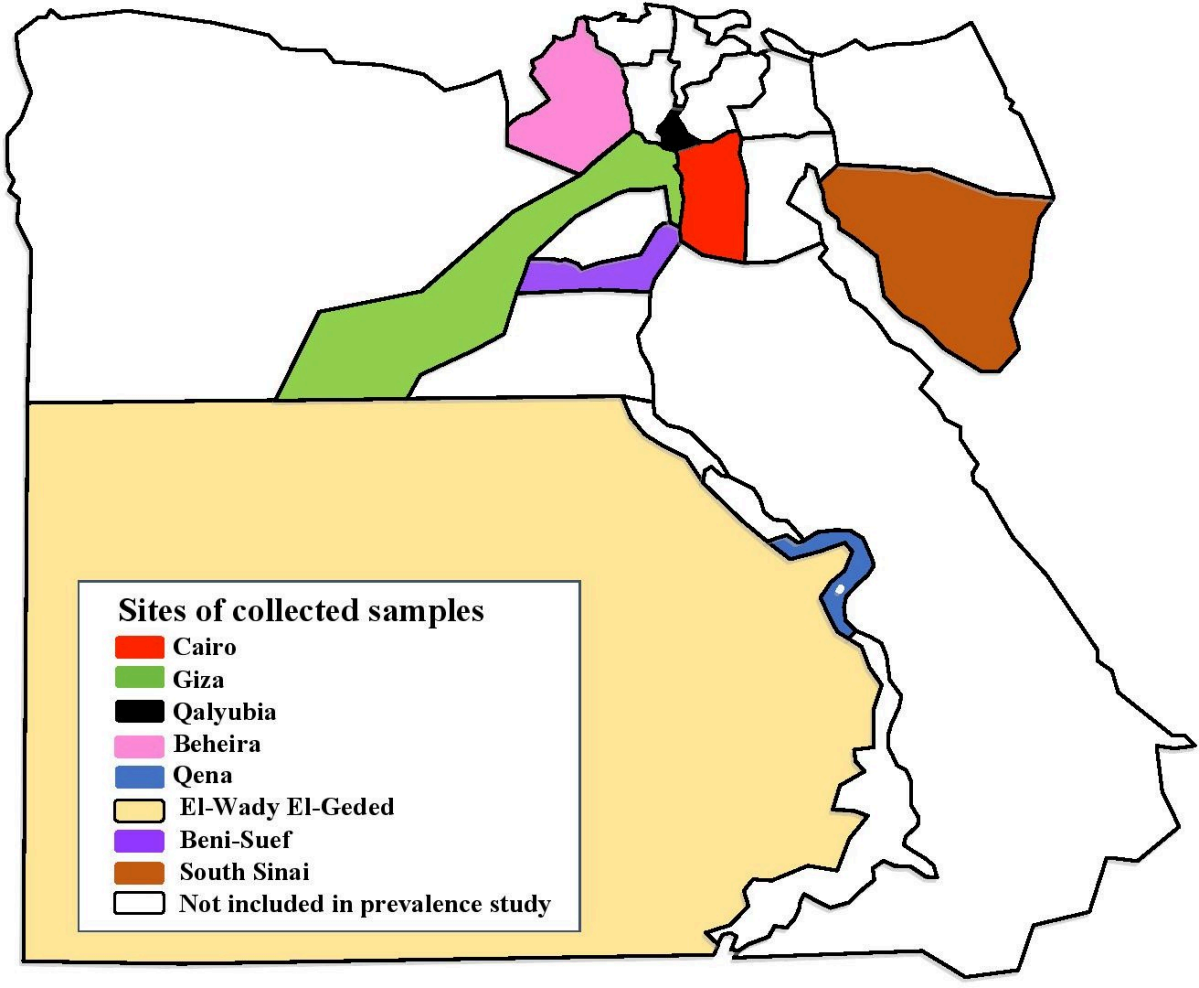
Map of Egypt showing the different provinces where the blood samples from different animal hosts were collected for our study. https://en.wikipedia.org/wiki/Governorates_of_Egypt and the picture has CC BY-SA 3.0.

We conducted a cross-sectional observational study with a total of 557 apparently healthy domestic animals (203 dogs, 149 camels, 88 cattle, 26 buffaloes, 58 sheep and 33 goats) using a convenience sampling strategy [81]. Animal blood samples were randomly collected from different provinces in Egypt between 2016 and 2018. The details of the sample locations were presented in Fig 1 and Table 1. For each animal host, 5 ml of blood was collected in a sterile EDTA tube using a sterile syringe and stored at -20°C for molecular purposes. The prevalence of infection of different pathogens by different animal hosts was calculated according to Thrusfield et al. [81].

**Table 1:** The information data of collected samples.

Provinces	Geographic coordinates	Animal Hosts	Locations	Numbers of Animals
**Cairo**	30° 03’ 45.47" N, 31° 14’ 58.81" E	Dog	Police Academy (El-Abbasia)	75
			Police Academy (El-Tagamoa)	67
			Police Academy (El-Dowaika)	61
		Camel	Police Academy (Gasr-El Swiss)	52
**Giza**	29° 58’ 27.00" N, 31° 08’ 2.21" E	Camel	Police Academy (El-Haram)	96
		sheep	households	5
		Goat	households	6
**Beni-Suef**	29° 03’ 60.00" N, 31° 04’ 60.00" E	Cattle	households	63
		Sheep	households	48
		Goat	households	20
		Buffalo	households	20
**Qalyubia**	30.41°N, 31.21°E	Cattle	households	2
		Buffalo	households	6
		Goat	households	2
**Sinai**	28° 32’ 13.79" N, 33° 58’ 14.39" E	Sheep	households	5
		Goat	households	5
		Camel	Free rearing	1
**El-Wady El-Geded**	24°32′44″N, 27°10′24″E	Cattle	households	11
**Qena**	26° 09’ 60.00" N, 32° 42’ 59.99" E	Cattle	households	10
**Beheira**	30.61°N, 30.43°E	Cattle	households	2

### DNA extraction

DNA was extracted from 200 μl of each blood sample using EZ1 DNA Blood Kit (Qiagen, Hilden, Germany) according to the manufacturer’s instructions. The extracted DNA was stored at -20°C until use for molecular screening.

### Screening of multiple pathogen DNA by qPCR

All samples were first screened for pathogen DNA by qPCR using genus-specific primers and probes targeting the 5.8S rRNA gene of piroplasms, the 23S rRNA gene of Anaplasmataceae, the *gltA* gene of *Rickettsia* sp., the 16S rRNA gene of *Borrelia* sp., the IS1111 of *C*. *burnettii*, BartoITS3 of *Bartonella* sp. and the pan-fil 28S rRNA gene of Filariidae. For positive Filariidae in dog samples, a triplex qPCR targeting Cox1 was used to detect *D*. *immitis*, *D*. *repenes* and *Ac*. *reconditum*. The sequence of primers and probes used in this study is showed in Table 2. The qPCR was preformed using a CFX 96 Real Time System (Bio-Rad Laboratories, Foster City, CA, USA). The total reaction volume of 20 μl included 10 μl of Eurogentec Master Mix Roche, 0.5 μl of each primer, 0.5 μl of FAM-labeled probe, 0.5 μl of UDG, 5 μl of DNA template, and 3 μl of DNAse- and RNAse-free water. Thermal cycling was performed according to the instructions provided by the manufacturer of the Master Mix PCR kit. To evaluate the PCR reaction, a positive control (pathogen DNA) and a negative control were added to each reaction. The sample was considered positive if the cycle threshold (Ct) was less than 35 Ct [82].

**Table 2:** Primers and probes used for qPCR, Standard PCR and sequencing in this study.

Microorganisms	Targeted gene	Primers F, R (5’-3’) and Probes S (6FAM–TAMRA)	Tm	References
Piroplasmida	5.8S rRNA 18S rRNA	5.8S-F5-AYYKTYAGCGRTGGATGTC 5.8S-R-TCGCAGRAGTCTKCAAGTC 5.8S-S-TTYGCTGCGTCCTTCATCGTTGT piro18SF1- GCGAATGGCTCATTAIAACA piro18SF4-TTTCAGMCTTGCGACCATACT piro18SF3-GTAGGGTATTGGCCTACCG piro18SR3-AGGACTACGACGGTATCTGA	- 58°C	**[135]**
Anaplasmataceae	23S rRNA (TtAna) 23S rRNA Ana-*rpoB*	TtAna-F-TGACAGCGTACCTTTTGCAT TtAna-R-GTAACAGGTTCGGTCCTCCA TtAna-S-CTTGGTTTCGGGTCTAATCC Ana23S-212F-GTTGAAAARACTGATGGTATGCA Ana23S-753R-TGCAAAAGGTACGCTGTCAC rpoB-F-GCTGTTCCTAGGCTYTCTTCGCGA rpoB-R-AATCRAGCCAVGAGCCCCTRTAWGG	- 55°C 52°C	**[24]**
*Rickettsia* sp.	*gltA* (RKNDO3) *gltA* *OmpB*	RKNDO3-F-GTGAATGAAAGATTACACTATTTAT RKNDO3-R-GTATCTTAGCAATCATTCTAATAGC RKNDO3-S-CTATTATGCTTGCGGCTGTCGGTTC CS2D-ATGACCAATGAAAATAATAAT CSEnd-CTTATACTCTCTATGTACA 120-M59-CCGCAGGGTTGGTAACTGC 120-607-AATATCGGTGACGGTCAAGG 120-1497- CCTATATCGCCGGTAATT	- 50°C 50°C	**[136]** **[137]** **[138]**
*Borrelia* sp.	Internal transcribed spacer 16S RNA (Bor ITS4) 16S rRNA	BorITS4-F-GGCTTCGGGTCTACCACATCTA BorITS4-R-CCGGGAGGGGAGTGAAATAG BorITS4-S-TGCAAAAGGCACGCCATCACC 16S-F-GCTGGCAGTGCGTCTTAAGC 16S-R-GCTTCGGGTATCCTCAACTC	- 57°C	**[139]**
*Coxiella burnetii*	Insertion Sequence (IS1111) Cox2 Cox5 Cox18	IS1111-F-CAAGAAACGTATCGCTGTGGC IS1111-R-CACAGAGCCACCGTATGAATC IS1111-S-CCGAGTTCGAAACAATGAGGGCTG Cox2-F-CAACCCTGAATACCCAAGGA Cox2-R-GAAGCTTCTGATATAGGCGGGA Cox5-F-CAGGAGCAAGCTTGAATGCG Cox5-R-TGGTATGACAACCCGTCATG Cox18-F-CGCAGACGAATTAGCCAATC Cox18-R-TTCGATGATCCGATGGCCTT	- 57°C	**[63]** **[140]**
*Bartonella* sp.	Internal transcribed spacer16S (BartoITS3)	BartoIRS3-F-GATGCCGGGGAAGGTTTTC BartoIRS3-R-GCCTGGGAGGACTTGAACCT BartoIRS3-S-GCGCGCGCTTGATAAGCGTG	-	**[141]**
*Filariidae*	Pan-fil 28S rRNA Triplex TaqMan *Cox1* SSU rRNA (18S)	qFil-28S-F-TTGTTTGAGATTGCAGCCCA qFil-28S-R-GTTTCCATCTCAGCGGTTTC qFil-28S-S-CAAGTACCGTGAGGGAAAGT Fil.COI.749-F-CATCCTGAGGTTTATGTTATTATTTT D.imm.COI.777-S-CGGTGTTTGGGATTGTTAGTG D.rep.COI.871-S-TGCTGTTTTAGGTACTTCTGTTTGAG Fwd.18S.631-TCGTCATTGCTGCGGTTAAA Rwd.1465-GGTTCAAGCCACTGCGATTAA	- 55°C	**[142]** **[143]** **[144]**

### Standard PCR and sequencing

All samples considered positive by qPCR were subjected to standard PCR and sequencing. Primers targeting 969 bp and 1200 bp region of the 16S rRNA gene, respectively, were used to identify *Piroplasma* and *Borrelia*. For the identification of Anaplasmataceae, standard PCR were performed with primers targeting a 520 bp fragment of the 23S rRNA gene. The positive samples with 23S rRNA gene were confirmed with *Anaplasma* genus-specific primers targeting the 525 bp fragment of the *rpoB* gene. *Rickettsia* genus-specific primers targeting the *gltA* gene were used and the positive samples were confirmed by the *ompB* gene. Moreover, multi-spacer typing (MST) for *C*. *burnetii* was performed by amplifying of three intergenic spacers (Cox2, Cox5 and Cox18). Identification of Filariidae was performed using 18S rRNA primers targeting 1155 bp. All primer sequences used in standard PCR and sequencing are listed in Table 2. All PCR reactions were performed in an Applied Biosystems 2720 Thermal Cycler model (Thermo Fisher Scientific Courtaboef, France) using AmpliTaq 360 Master Mix (Thermo Fisher Scientific Courtaboef, France) according to the manufacturer’s recommendations. Negative and positive controls were included in each reaction. PCR products were visualized by electrophoresis on a 1.5% agarose gel stained with Syper Safe stain (Invitrogen, USA) and analyzed using Lab Image software (BioRad, Marnes-La-Coquette, France).

PCR products were purified using NucleoFast 96 PCR plates (Macherey Nagel, EURL, Hoerdt, France), according to the manufacturer’s recommendation. The purified PCR products were sequenced using the Big Dye Terminator Cycle Sequencing Kit (Perkin Elmer Applied Biosystems, Foster City, CA, USA) with an ABI automated sequencer (Applied Biosystems). The sequences obtained were assembled and edited using ChromasPro software (ChromasPro 1.7, Technelysium Pty Ltd., Tewantin, Australia), and the corrected sequences were compared with the sequences available in GenBank by BLAST (https://blast.ncbi.nlm.nih.gov/Blast.cgi).

### Phylogenetic analyses

Multiple sequence alignments were performed between the obtained sequences and other reference sequences in GenBank using CLASTAL W in MEGA software version X [83]. Phylogenetic trees were inferred using the Maximum-Likelihood method and Tamura-Nei model with 500 bootstrap replicates in MEGA X software [83,84].

## Results

In this study, all samples (557) were screened by qPCR. None of the animals were positive for *Bartonella* sp., while different animal hosts were positive for piroplasms, *Anaplasma* sp., *Rickettsia* sp., *Borrelia* sp., *C*. *burnettii* and *Filaria* sp (Table 3).

**Table 3:** The prevalence of pathogens in animals by PCR.

Animal Hosts	No. of examined Animals (Total = 557)	Pathogens amplified	No. of infected Animals (%)
**Cattle**	88	**Piroplasmida** *T*. *annulata* *Ba*. *bigemina* **Anaplasmataceae** *An*. *marginale* *An*. *centrale* *An*. *ovis* *An*. platys-like ***Borrelia* sp.** *Bo*. *theileri* **Co-infection :** *An*. *marginale* + *T*. *annulata* *An*. *marginale* + *Bo*. *theileri* *An*. *centrale* + *T*. *annulata* *An*. *platys*-like + *Ba*. *bigemina*	**15/88 (17%)** 14/88 (15.9%) 1/88 (1.1%) **25/88 (28.4%)** 18/88 (20.4%) 1/88 (1.1%) 3/88 (3.4%) 3/88 (3.4%) **3/88 (3.4%)** **5/88 (5.7%)** 2/88 (2.3%) 1/88 (1.1%) 1/88 (1.1%) 1/88 (1.1%)
**Buffalo**	26	**Piroplasmida** *T*. *ovis* **Anaplasmataceae** *An*. platys-like	**2/26 (7.7%)** **2/26 (7.7%)**
**Sheep**	58	**Piroplasmida** *T*. *ovis* **Anaplasmataceae** *An*. *marginale* *An*. *ovis* *An*. platys-like ***Borrelia* sp.** *Bo*. *Theileri* ***Coxiella burnetii*** **Co-infection :** *An*. *platys*-like + *Bo*. *theileri*	**5/58 (8.6%)** **4/58 (6.9%)** 2/58 (3.4%) 1/58 (1.7%) 1/58 (1.7%) **2/58 (3.4%)** **1/58 (1.7%)** **1/58 (1.7%)**
**Goat**	33	***Coxiella burnetii***	**1/33 (3%)**
**Camel**	149	**Anaplasmataceae** *An*. *marginale* *An*. platys *An*. platys-like **Filariidae** *S*. *digitate*	**10/149 (6.7%)** 1/149 (0.7%) 1/149 (0.7%) 8/149 (5.4%) **1/149 (0.7%)**
**Dog**	203	**Piroplasmida** *Ba*. *canis* **Anaplasmataceae** *An*. *platys* ***Rickettsia* sp.** *Rickettsia africae*-like **Filariidae** *D*. *repens* *Ac*. *reconditum* **Co-infection :** *R*. *africae*-like + *Anaplasma*	**1/203 (0.5%)** **7/203 (3.4%)** **3/203 (1.5%)** **3/203 (1.5%)** 2/203 (1%) 1/203 (0.5%) **1/203 (0.5%)**

**Fig 2:**
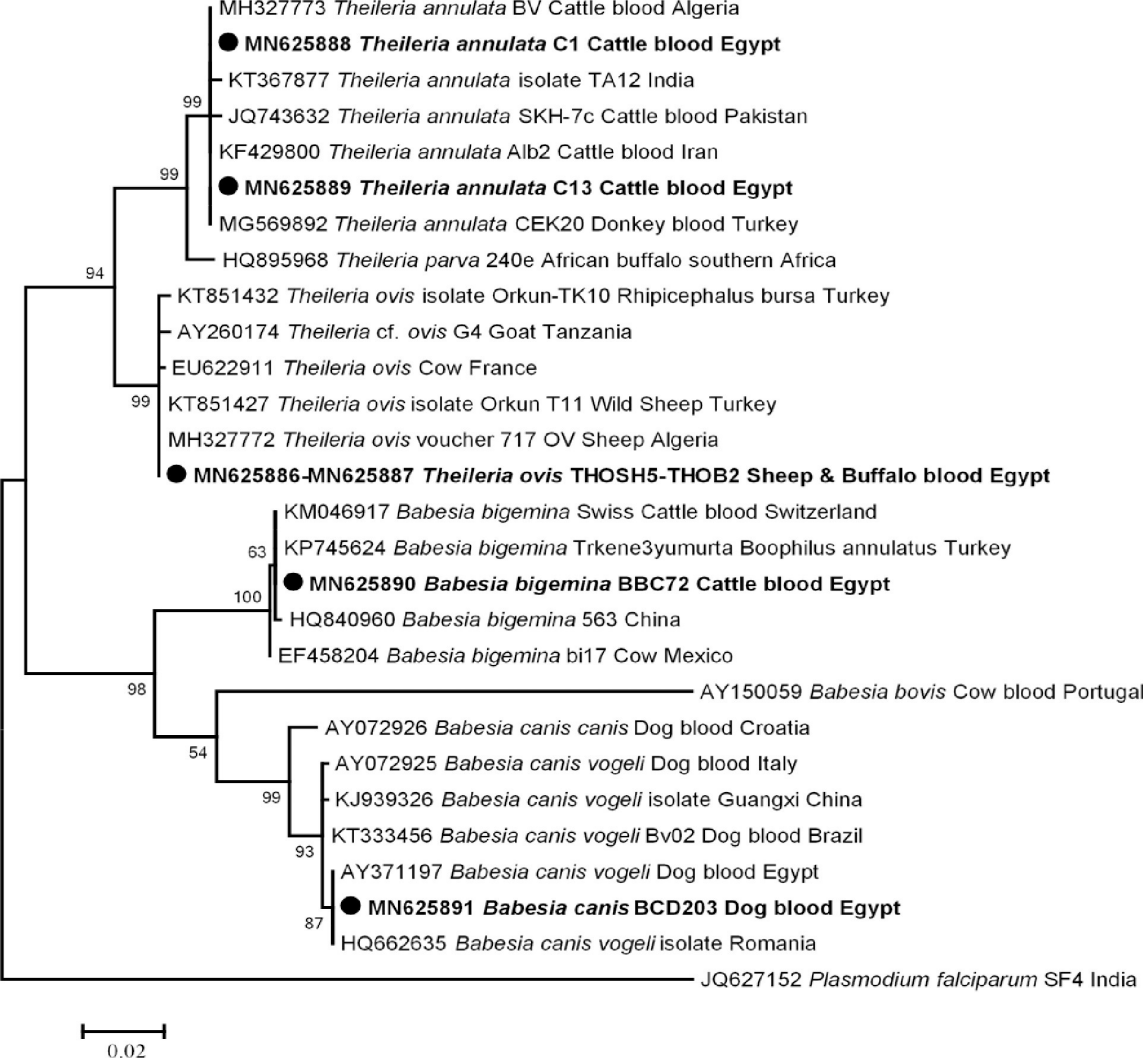
18S rRNA based phylogenetic analysis of genotypes identified in this study. Phylogenetic tree highlighting the position of *Theileria* sp. and *Babesia* sp. in the present study (**Bold**) related to other *Theileria* sp. and *Babesia* sp. available in GenBank. The sequence of 18S rRNA were aligned using CLUSTAL W and phylogenetic inferences were constructed in MEGA X using Maximum Likelihood based on Tamura-Nei Model for nucleotide sequences with 500 bootstrap replicates. There was a total of 1066 positions in the final dataset. The scale bar represents a 2% nucleotide sequence divergence.

Fifty of 557 (8.9%) animal hosts were positive for piroplasms based on 5.8S rRNA qPCR system. Standard PCR and sequencing based on 18S rRNA gene succeeded in amplifying and identifying two *Theileria* sp.; *T*. *annulata* in cattle (14/88), *T*. *ovis* in sheep and buffaloes (5/58 and 2/26, respectively) and two *Babesia* sp.; *Ba*. *bigemina* in cattle (1/88) and *Ba*. *canis* in dogs (1/203). However, camels and goats were free of Piroplasmida DNA. The overall prevalence of piroplasmoses in different animal hosts was 23/557 (4.1%) as it was 17% in cattle, 8.6% in sheep, 7.7% in buffaloes and 0.5% in dogs. In our study, BLAST analysis revealed that cattle were positive for *T*. *annulata* and *Ba*. *bigemina*, including two genotypes of *T*. *annulata*, one genotype in 13 cattle with 100% (910/910) similarity to those of *T*. *annulata* detected in donkey blood in Turkey (GenBank: MG569892), a new genotype in one cattle with 99% (908/910) identity to the same reference dataset, and a new genotype of *Ba*. *bigemina* in one cattle with 99% (865/866) identity to those of *Ba*. *bigemina* detected in cattle blood from Switzerland (GenBank: KM046917). Similarly, we found that 5 sheep and 2 buffaloes were positive for a genotype of *T*. *ovis* with 100% (897/897) identity to *T*. *ovis* detected in wild sheep from Turkey (GenBank: KT851427). Finally, we identified *Ba*. *canis* in a dog with 100% (884/884) similarity to those of *Ba*. *canis vogeli* detected in a dog from Egypt (GenBank: AY371197). The phylogenetic tree of these genotypes was illustrated in Fig 2.

**Fig 3:**
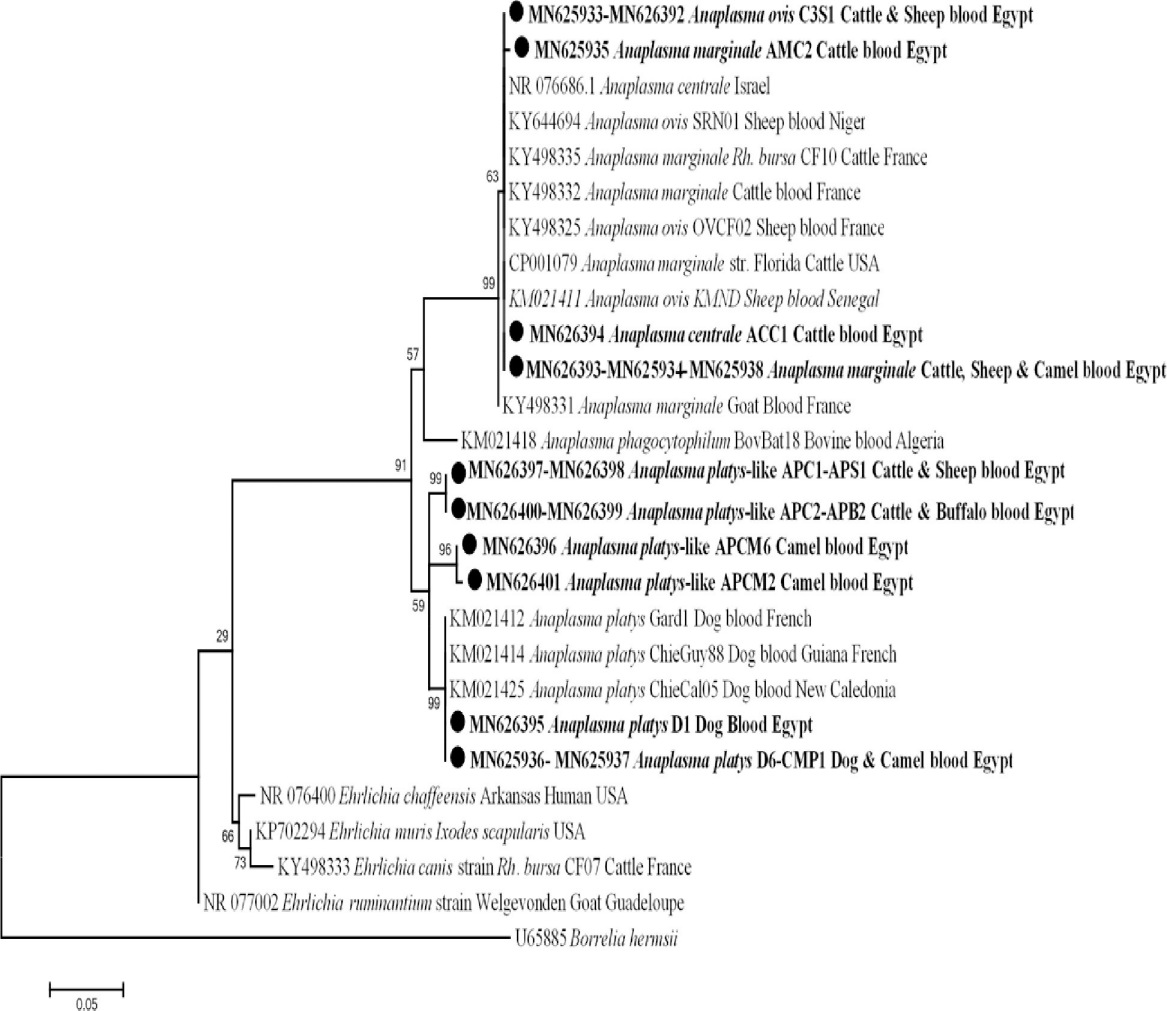
23S rRNA based phylogenetic analysis of genotypes identified in this study. Phylogenetic tree highlighting the position of *Anaplasma* sp. in the present study (Bold) related to other *Anaplasma* sp. and *Ehrlichia* sp. available in GenBank. The sequence of 23S rRNA were aligned using CLUSTAL W and phylogenetic inferences were constructed in MEGA X using Maximum Likelihood based on Tamura-Nei Model for nucleotide sequences with 500 bootstrap replicates. There was a total of 432 positions in the final dataset. The scale bar represents a 5% nucleotide sequence divergence.

For Anaplasmataceae, 172 out of 557 (30.9%) animal hosts were positive for anaplasmoses by 23S rRNA qPCR system. Based on the 23S rRNA gene, only 87 out of 557 animal hosts were successfully amplified by standard PCR, consequently, sequencing identified only 48 out of 557. The overall prevalence of anaplasmoses in different animals was 8.6%, with 28.4% in cattle (25/88), 6.9% in buffaloes (4/58), 7.7% in sheep (2/26), 6.7% in camels (10/149) and 3.4% in dogs (7/203), while goats were free of *Anaplasma* DNA. BLAST analysis revealed that cattle, sheep and camel were positive *An*. *marginale*, including two different genotypes of *An*. *marginale*, the first originated from sixteen cattle, two sheep and one camel with 100% (455/455) similarity to those of *An*. *marginale* detected in *Rh*. *bursa* collected from cattle in France (GenBank KY498335), and another new genotype was detected in two cattle with 99% (454/455) identity to the same reference dataset (GenBank KY498335). Moreover, one case of cattle was positive for *An*. *centrale* with 100% identical to *An*. *centrale* strain Israel (GenBank NR076686). From cattle and sheep, a genotype of *An*. *ovis* was identified with 100% (454/454) similarity to *An*. *ovis* in sheep blood from Niger (GenBank KY644694). We found that dogs and camels were positive for *An*. *platys*, including two different genotypes of *An*. *platys*, one genotype from six dogs and one camel with 100% (458/458) identity to *An*. *platys* in dog blood from France (GenBank KM021425) and another genotype from one dog with 100% (458/458) homology to *An*. *platys* in dog blood from France (GenBank KM021414). Finally, from cattle, buffaloes, sheep and camels, a new potential *Anaplasma* sp. was identified including, four different genotypes of this *Anaplasma* sp., the first genotype from six camels, the second from two camels, the third from one cattle and one sheep and the last from two cattle and two buffaloes with 98% (450/458), 98% (448/458), 98% (447/458) and 97% (446/458) similarity, respectively, to *An*. *platys* in dog blood from France (GenBank KM021414). Sequence analysis of this *Anaplasma* species revealed that this species has a homology score below 99% (more than 10 nucleotides different) and are closely related to *An*. *platys*, that means these sequences could be considered as potential new species of *Anaplasma* and can be called as *An*. *platys*-like. The phylogenetic tree showed that the new potential *Anaplasma* sp. in two separates and well-supported branches (bootstraps 99 and 96) belong to the cluster of *An*. *platys* (Fig 3).

**Fig 4:**
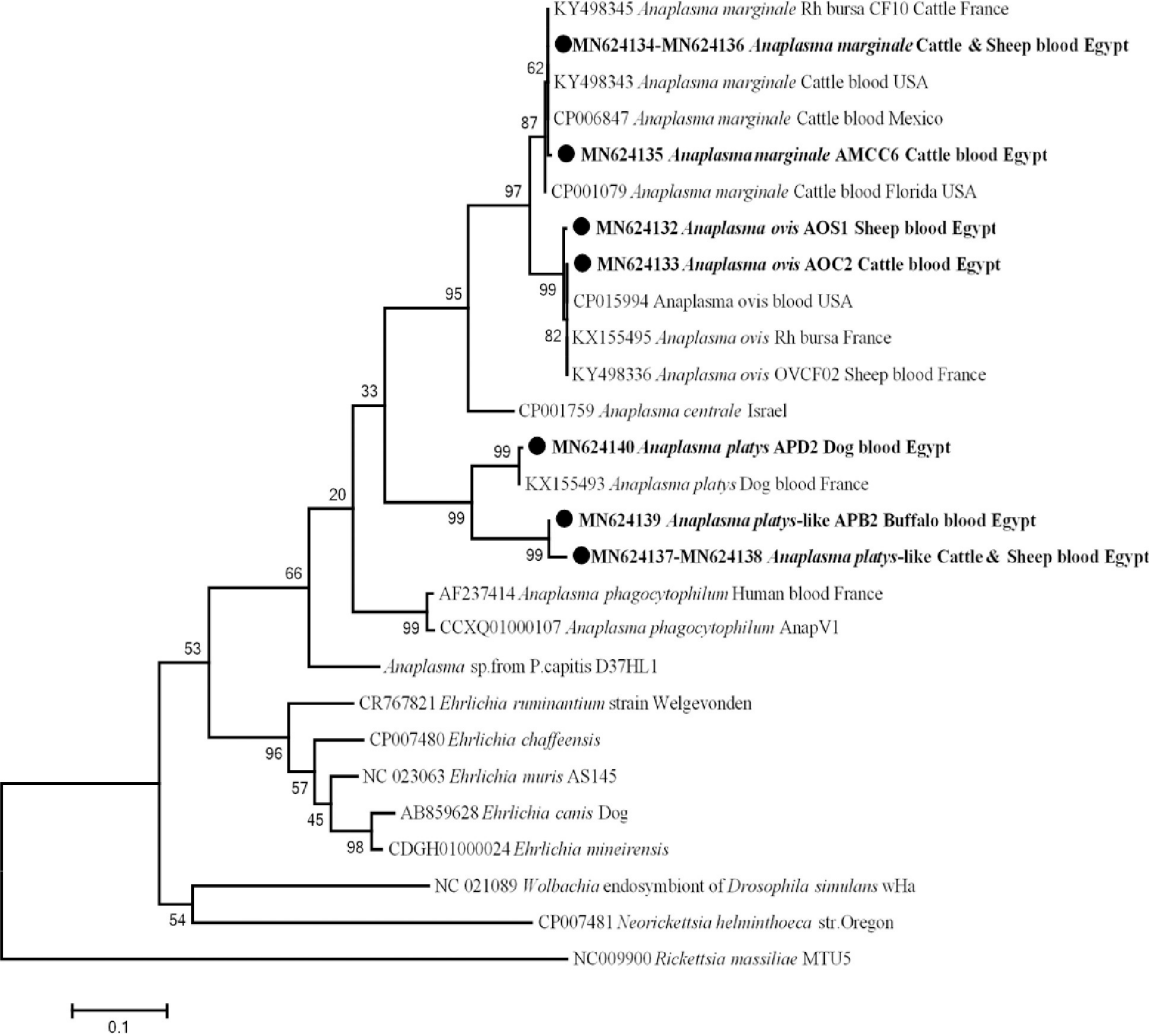
*rpoB* gene based phylogenetic analysis of genotypes identified in this study. Phylogenetic tree highlighting the position of *Anaplasma* sp. in the present study (**Bold**) related to other *Anaplasma* sp. and *Ehrichia* sp. available in GenBank. The sequence of *rpoB* gene were aligned using CLUSTAL W and phylogenetic inferences were constructed in MEGA X using Maximum Likelihood based on Tamura-Nei Model for nucleotide sequences with 500 bootstrap replicates. There was a total of 534 positions in the final dataset. The scale bar represents a 10% nucleotide sequence divergence.

To better characterize different *Anaplasma* genotypes, *rpoB* genus-specific PCR primers were applied and 23 good quality sequences were identified. The result revealed that, 12 cattle and one sheep were positive for a genotype of *An*. *marginale* with 100% (487/487) homology with *An*. *marginale* in *Rhipicephalus bursa* from France (KY498345), and another genotype of *An*. *marginale* from one cattle with 99% (486/487) similarity with the same reference dataset. We also identified that cattle and sheep were positive for *An*. *ovis*, one genotype was found in two cattle and another in a sheep with 100% (489/489) and 99% (487/489) identical to those of *An*. *ovis* in sheep blood from Niger (GenBank KY644695), respectively. From dogs, we identified a new genotype of *An*. *platys* obtained from two dogs with 99% (488/489) homology to *An*. *platys* in dog blood from France (GenBank KX155493). Finally, from cattle, buffaloes and sheep, a new potential species of *Anaplasma*. was identified, its sequences had a homology score of less than 90%, confirming that these sequences are likely to be a new potential species of *Anaplasma* (like 23S rRNA gene). The only two different genotypes (one from two buffaloes and another from a cattle and a sheep) showed a low identity of 89% (432/486) and 88% (431/486), respectively, with *An*. *platys* in dog blood from France (GenBank KX155493), while identification of the genotype derived from camels failed. Phylogenetic analysis revealed a new potential *Anaplasma* sp. (*An*. *platys*-like) in a separate and well-supported branch (bootstraps 99) with the same clade belonging to *An*. *platys* (Fig 4).

**Fig 5:**
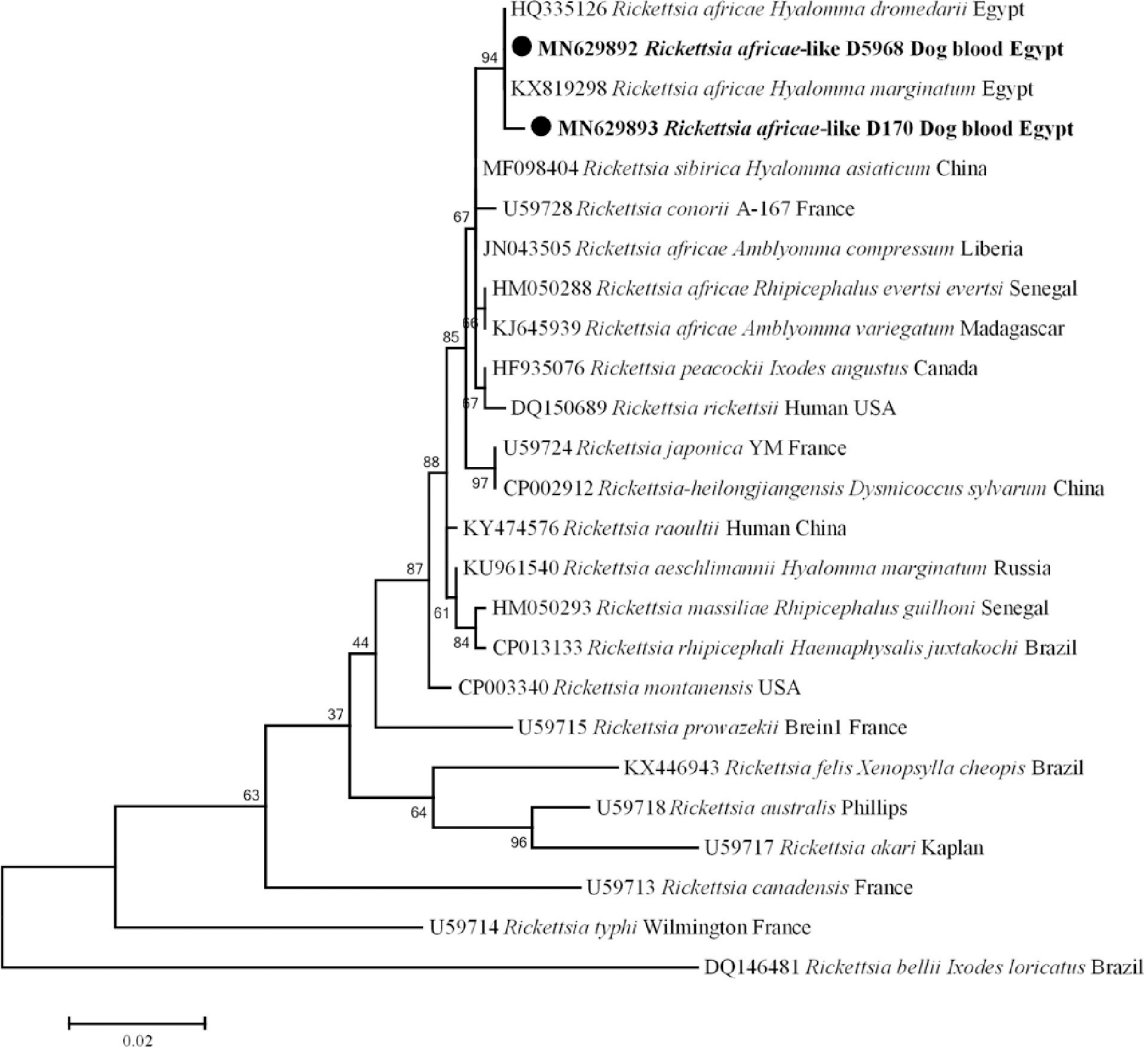
*gltA* gene based phylogenetic analysis of genotypes identified in this study. Phylogenetic tree highlighting the position of *Rickettsia* sp. in the present study (**Bold**) related to other *Rickettsia* sp. available in GenBank. The sequence of *gltA* gene were aligned using CLUSTAL W and phylogenetic inferences were constructed in MEGA X using Maximum Likelihood based on Tamura-Nei Model for nucleotide sequences with 500 bootstrap replicates. There was a total of 728 positions in the final dataset. The scale bar represents a 2% nucleotide sequence divergence.

Rickettsial infection was detected by qPCR targeting *gltA* gene in dogs (3/557; 0.54%); the other animal hosts were free of rickettsiosis. To identify *Rickettsia* sp., standard PCR and sequencing were performed using *gltA* gene, and it was possible to amplify a 728 bp fragment of this gene from these three positive samples. A BLAST search of the obtained sequences with those in GenBank revealed that two different genotypes, one genotype was 100% (728/728) identical with *R*. *africae* previously detected in *H*. *dromedarii* from Egypt (GenBank: HQ335126), and the other sequence had 99% (726/728) identity with the same reference. Moreover, *ompB* gene was used to confirm the detection of *R*. *africae*-like infection in dogs. Based on the BLAST search, the sequences obtained from dogs were identified as *R*. *africae* (GenBank: MN629894) and showed (757/758) 99% similarity with the reference stain of *R*. *africae* detected in a traveler returning from Tanzania (GenBank: KU721071). The phylogenetic tree of these *R*. *africae*-like in dogs based on *gltA* was shown in Fig 5.

**Fig 6:**
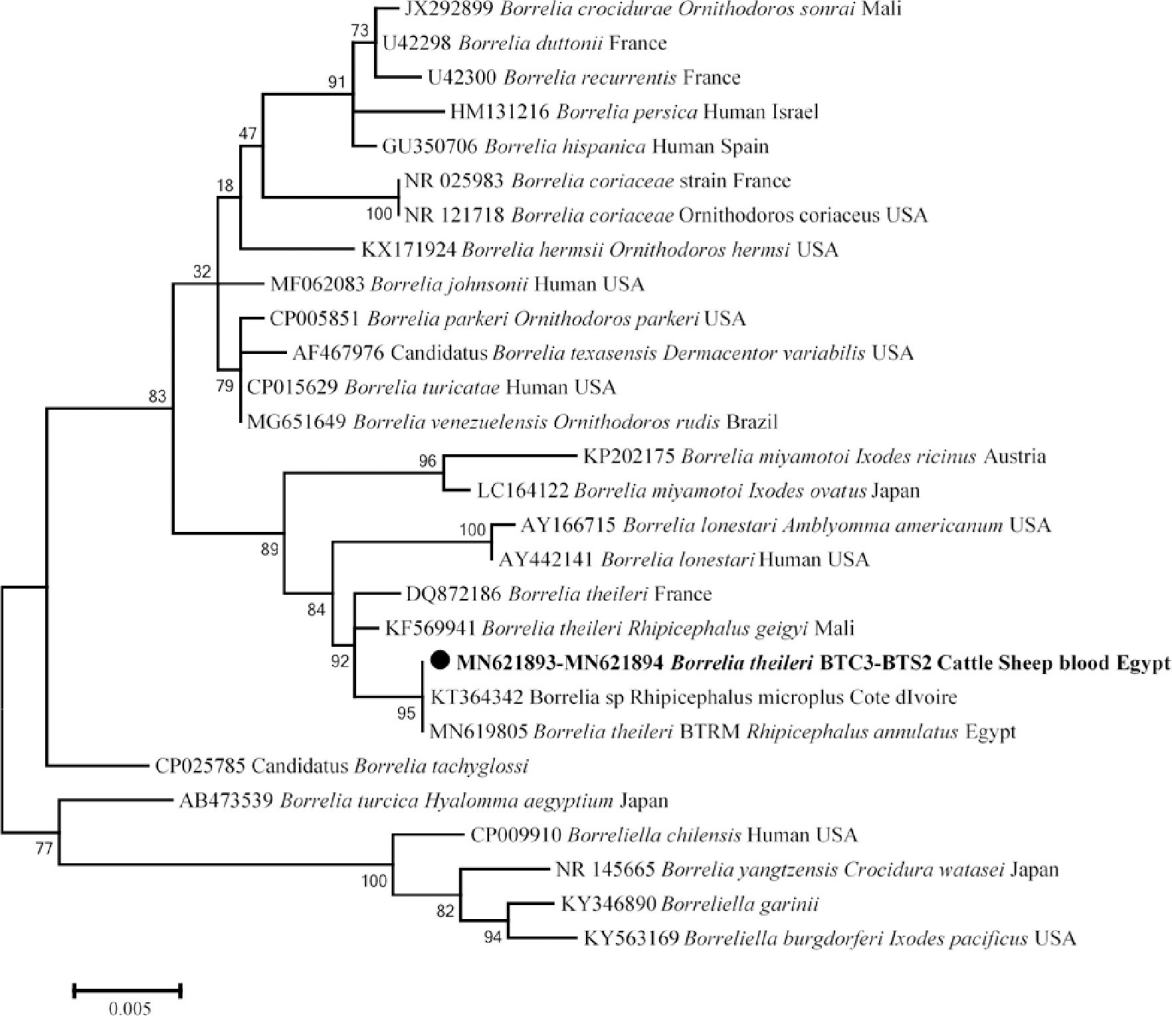
16S rRNA based phylogenetic analysis of genotypes identified in this study. Phylogenetic tree highlighting the position of *Borrelia* sp. in the present study (**Bold**) related to other *Borrelia* sp. available in GenBank. The sequence of 16S rRNA were aligned using CLUSTAL W and phylogenetic inferences were constructed in MEGA X using Maximum Likelihood based on Tamura-Nei Model for nucleotide sequences with 500 bootstrap replicates. There was a total of 1142 positions in the final dataset. The scale bar represents a 5% nucleotide sequence divergence.

Screening of *Borrelia* sp. in all animal hosts we found that 3 cattle and 2 sheep were positive for *Borrelia* sp. (5/557; 0.9%). Standard PCR and sequencing using 16S rRNA gene identified it as *Bo*. *theileri*. Alignment of five obtained sequences of *Borrelia* sp. from our samples revealed that all sequences were identical to each other. Furthermore, comparison of the obtained sequences with sequences from the GenBank database showed that 1139/1143 (99%) identity with *Bo*. *theileri* detected in *Rh*. *geigyi* in Mali (GenBank: KF569941). The phylogenetic position of this new *Bo*. *theileri* genotype was shown in Fig 6.

Two out of 557 (0.36%) blood samples from one sheep and one goat tested positive for *C*. *burnetii* DNA by qPCR targeting IS1111. MST genotyping was performed using Cox2, Cox5 and Cox18, with only Cox2 successfully identified and the other spacers failing amplification. A BLAST search for the two sequences obtained showed that (351/351) 100% identity with the reference sequences of *C*. *burnetii* recorded in GenBank.

**Fig 7:**
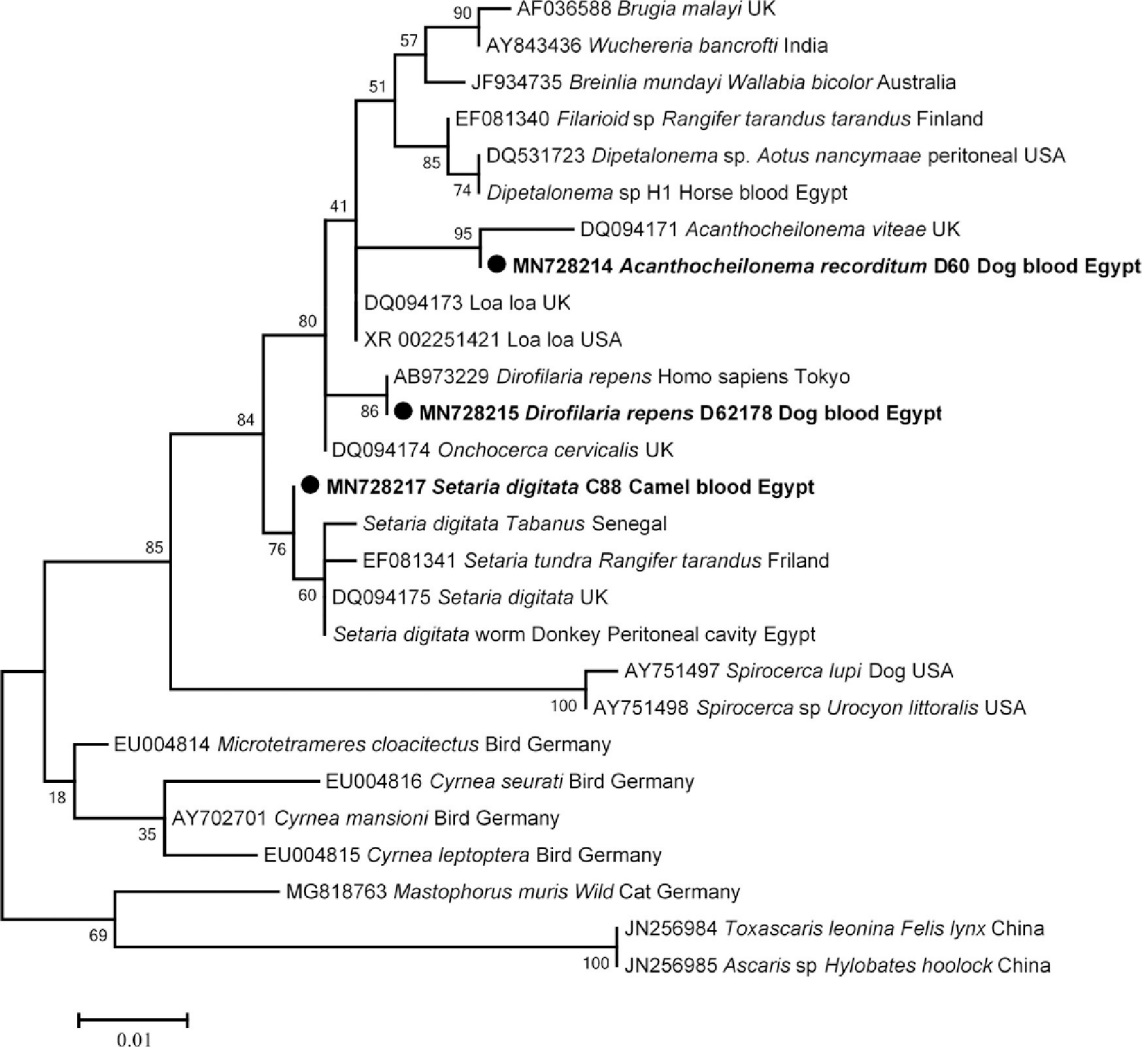
18S rRNA based phylogenetic analysis of genotypes identified in this study. Phylogenetic tree highlighting the position of *Filaria* sp. in the present study (**Bold**) related to other *Filaria* sp. available in GenBank. The sequence of 18S rRNA were aligned using CLUSTAL W and phylogenetic inferences were constructed in MEGA X using Maximum Likelihood based on Tamura-Nei Model for nucleotide sequences with 500 bootstrap replicates. There was a total of 1110 positions in the final dataset. The scale bar represents a 10% nucleotide sequence divergence.

Concerning Filariidae, four out of 557 (0.7%) animal hosts collected from three dogs and one camel tested positive for *Filaria* sp. DNA. By BLAST analyses, two dogs were found to have *D*. *repens* with 100% identity to those of *D*. *repens* previously detected in a Japanese woman returned from Europe (GenBank AB973229), and another sequence obtained from one dog showed 99% (1114/1119) similarity to *Ac*. *viteae* (GenBank: DQ094171). Moreover, *S*. *digitata* with (1107/1111) 99% identity to *S*. *digitata* from UK (GenBank: DQ094175) was found in a camel. The phylogenetic analysis of these *Filaria* sp. was constructed and presented in Fig 7.

Finally, seven of different animal hosts were positive for more than one vector-borne pathogen (co-infections; 7/557; 1.3%). In cattle, five co-infections were observed (5/88; 5.7%) as *An*. *marginale* plus *T*. *annulata* (2/88; 2.3%), *An*. *marginale* plus *Bo*. *theilerii* (1/88; 1.1%), *An*. *centrale* plus *T*. *annulata* (1/88; 1.1%) and *An*. *platys*-like with *Ba*. *bigemina* (1/88; 1.1%). Moreover, one co-infection in sheep was recorded as *An*. *platys*-like plus *Bo*. *theilerii* (1/58; 1.7%) and one case in dogs *R*. *africae*-like with *Anaplasma* (1/203; 0.5%) (Table 3).

## Discussion

The sustainable and economic progress of developing countries depends mainly on domestic animal resources, as they provide vital food, draught power and manure for crop production, and generate income [85]. However, animal-associated diseases, especially, VBDs are a global burden [2]. Recently, the spectrum of VBDs affecting animals has expanded and the attention of clinicians and veterinarians is growing. Therefore, the diagnosis of VBDs is crucial to develop the epidemiological mapping of these diseases and this can be achieved through the advances in molecular biology [86].

Concerning piroplasmoses, the overall prevalence among animal hosts was 4.1%, including the highest prevalence among cattle 17%, then sheep 8.6%, buffaloes 7.7% and dogs 0.5%. Based on the 18S rRNA gene, two genotypes of *T*. *annulata* was detected in cattle from different provinces (El-Wady El-Geded, Beni-Suef, Qena and Beheira) and one case of *Ba*. *bigemina* was detected in cattle from Beni-Suef. In accordance to our results, many studies reported the high prevalence of *T*. *annulata* compared to other piroplasms in cattle from different provinces in Egypt [87–89]. In the current study, we observed that the majority of cases (10 out of 15) were detected in cattle from El-Wady El-Geded province that in accordance with Al-Hosary et al. [89], who stated that the prevalence of *T*. *annulata* in cattle from El-Wady El-Geded province was 63.6%. This finding might be due to the climate in this province, which is dry and sunny throughout the year, which is conducive to tick activity [89]. Likewise, we identified *T*. *ovis* in sheep from Giza and Beni-Suef and buffaloes from Beni-Suef. In Egypt, there are few studies reporting *T*. *ovis* in sheep [90] and buffaloes [91]. In parallel, a recent study reported that *T*. *ovis* was detected in sheep from Menoufia and El-Wady El-Geded province [92], implying that this pathogen is widespread in sheep throughout Egypt. Finally, we detected one case of *Ba*. *canis* in a dog from Cairo province with 100% identity with *Ba*. *canis vogeli* detected in a dog from Egypt (GenBank: AY371197). Canine babesiosis is distributed worldwide and was later detected in Egypt by Passos et al. [93] and Salem and Farag [94]. In Africa, *Ba*. *canis vogeli* has been detected in different regions such as South Africa [95], Tunisia [96] and Côte d’Ivoire [80].

Family Anaplasmataceae was known to cause human and animal diseases, is transmitted by ticks and has a worldwide distribution [26,97]. In the current study, the overall prevalence of anaplasmosis was 30.9% (172/557) by qPCR, while we obtained only 48 samples with good quality sequences, possible due to the higher sensitivity of qPCR compared to standard PCR or due to the co-infection with family Anaplasmataceae. The overall infection rate of *An*. *marginale* was 3.8% (21/557) in cattle, sheep and camels from different localities (Beni-Suef, Qena, El-Wady El-Geded and Cairo). In Egypt, *An*. *marginale* was first mentioned in the national report in 1966, after which the disease was reported in numerous provinces [32–34,98]. Several studies reported endemicity of *An*. *marginale* in cattle [16,28,31–34], buffaloes [30] and camels [29]. However, *An*. *marginale* was detected for the first time in sheep. To our knowledge, *An*. *marginale* has not yet been described in sheep. For the first time, *An*. *centrale* was detected in a bovine from El-Wady El-Geded province, Egypt. *Anaplasma centrale* is closely related to *An*. *marginale* but less pathogenic, so it has been used as a live vaccine to protect against bovine anaplasmosis [99,100]. We also found that sheep and cattle from Beni-Suef province (upper Egypt) were positive for *An*. *ovis* with a prevalence rate of 0.7% (4/557). To the best of our knowledge, *An*. *ovis* has never been detected in cattle and sheep in Egypt. In parallel, a recent study reported that *An*. *ovis* was detected in sheep in Menoufia province (one of Delta provinces) [34], implying that this pathogen is widespread in cattle and sheep throughout Egypt. *Anaplasma ovis* is the etiological agent of ovine anaplasmosis in small ruminants and causes mild and subclinical infections [23]. In Africa, some studies reported *An*. *ovis* in sheep from Tunisia [101], Senegal [25] and Algeria [102,103], and in cattle from Algeria [103]. In addition, we found that dogs from Cairo and a camel from Giza province were positive for two genotypes of *An*. *platys*, with an infection rate of 1.4% (8/557). In Egypt, *An*. *platys* was never molecularly identified in dogs and camels. Later, Loftis et al, [51] detected *An*. *platys* in ticks collected from dogs. *Anaplasma platys* is the causative agent of canine anaplasmosis, which causes severe thrombocytopenia in dogs [104]. Interestingly, we detected that cattle, buffaloes and sheep from Beni-Suef province and camels from Giza and Cairo provinces were positive for a new potential *Anaplasma* sp. with a prevalence rate of 2.5% (14/557). This probably new species was genetically related to canine *An*. *platys*, which is why it was commonly referred to as *An*. *platys*-like. This *An*. *platys*-like genotype has never been detected in Egypt, except in a recent study where *An*. *platys*-like bacterium was detected only in cattle in Menoufia province [34], implying that this new potential pathogen circulates between different animal hosts (excluding dogs that seem to be susceptible for a type *An*. *platys* only) and different provinces in Egypt. Later, *An*. *platys*-like was detected in various animal hosts such as cattle in Italy [105], Algeria [106] and Tunisia [107], camels in Tunisia [108,109] and sheep and goats in South Africa [110] and Senegal [25]. Various *Anaplasma* sp. were identified by the 23S RNA gene and which further confirmed by the *rpoB* gene.

Rickettsioses are VBDs of humans and animals and are mainly transmitted by ticks [35]. In Africa, the human pathogens *R*. *africae*, *R*. *aeschlimannii*, *R*. *conorii and R*. *massiliae* have been identified in ticks and animals [39–41]. In our study, rickettsial DNA was detected in dogs from Capital Cairo with a prevalence of 1.5% (3/203) in dogs. Phylogenetic analysis showed that our genotypes (*R*. *africae*-like) clustered in a separate and well-supported branch (bootstraps 94) with *R*. *africae* previously detected in Egypt **(**Fig 5) [53]. To the best of our knowledge, *R*. *africae* has not been previously detected in dogs anywhere in the world. Thus, this is the first detection of *R*. *africae*-like pathogens in dog anywhere in the world. African tick-bite fever, a benign disease with severe complications in elderly populations, and transmitted mainly in the south and West Africa by *Amblyomma variegatum* [35,111]. Likewise, *R*. *africae* was identified in other tick genera as *Hyalomma* sp. [42,53,54,112] and in *Rh*. *sanguineus* **(**the most common tick parasitizing dogs) [113].

Relapsing fever borrelioses caused by group of the spirochete group *Borrelia* sp. and is transmitted by soft and hard ticks [57]. In the present study, we identified *Bo*. *theileri* in bovine and ovine blood for the first time in Beni Suef province, Egypt, with an overall prevalence of 0.9% (5/557). Alignment of five sequences obtained revealed that there is a new potential genotype of *Bo*. *theileri* circulating between cattle and sheep in Beni-Suef province, which is 99% identical to *Bo*. *theileri* found in *Rh*. *geigyi* in Mali [58]. *Borrelia theileri* is considered one of the relapsing fever borreliae and the etiological agent of bovine borreliosis in cattle, transmitted by hard ticks, mainly *Rhipicephalus* sp. [114]. In Egypt, *Bo*. *theileri* was reported in *Rh*. *annulata* collected from donkeys in the same province [115]. Later, *Bo*. *theileri* was also detected in *Rh*. *annulata* in Egypt [62]. Recently, some studies have detected *Bo*. *theileri* in cattle such as Argentina [116] and Cameroon [117]. Similarly, *Bo*. *theileri* has been detected in the blood of sheep in Algeria [102]. It appears that, *Bo*. *theilerii* is not exclusively pathogenic to cattle.

Q fever is a tick-borne disease that is a major public health concern [65]. The infection in human manifests as acute or chronic febrile disease often associated with endocarditis and abortion [65]. In Egypt, Q fever was first detected in a high-risk group of cattle farmers [118]. Later, many reports demonstrated the prevalence of the disease in goats, sheep, cattle and camels [67–72,119,120]. In this study, the overall prevalence of Q fever in sheep and goats from Sinai province is 0.3% (3% in goats and 1.7% in sheep). This result was in accordance with Abdel-Moein and Hamza [71] who reported an overall prevalence of Q fever of 0.9% and 3.4% in goats. PCR and sequencing amplified only Cox2 with a 100% match with the *C*. *burnetii* reference recorded in GenBank, while genotyping and sequencing of the positive samples with other spacers (Cox5 & Cox18) failed. This result can be explained by the fact that the high sensitivity of qPCR can detect low DNA concentrations and the lower prevalence of *C*. *burnetii* in blood is lower than feces and urine [121,122].

For filarial infections, we detected four cases of filarial infection with an overall prevalence of 0.7%, 1.5% (3/203) in dogs and 0.7% (1/148) in camels. In dogs from the capital Cairo, we identified two different species of Filariidae as *D*. *repens* and *Acanthocheilonema* sp. *Acanthocheilonema viteae* is the filarial nematode of rodents, while *Ac*. *reconditum* is the etiological agent of filariasis in dogs. Also, there is no sequence of *Ac*. *reconditum* for the 18S rRNA gene in GenBank. Therefore, we suspect that the identified species is, however, *Ac*. *reconditum*. Therefore, this is the first report of *D*. *repens* and *Ac*. *reconditum* in dogs in Egypt. Subcutaneous dirofilariasis of domestic dogs is caused by *D*. *repens* and is common in Africa, Asia and Europe [123]. It is a mosquito-borne nematode that is a public health problem [124]. *Acanthocheilonema reconditum* colonizes the peritoneal cavity and adipose tissue and can cause skin lesions with allergy and is transmitted by fleas and biting lice [78,125,126]. In Africa, some studies reported microfilariae of *Ac*. *reconditum* in dogs in South Africa [127], Côte d’Ivoire [80]. Moreover, a camel from Giza province was positive for filarial nematodes, and was identified as *S*. *digitata*. To our knowledge, *S*. *digitata* has not been previously detected in camels. *Setaria digitata* is the natural filarial nematode of the Bovidae and the adult worm is resident in the peritoneal cavity [128,129]. Accidental transmission of *S*. *digitata* to unnatural hosts such as horses, donkeys, sheep and goats causes worrisome pathological problems such as corneal opacity and blindness [74,130,131–133].

Finally, we reported 1.3% (7/557) co-infections in animals, with the highest percentage in cattle 5.7% (5/557). Co-infection in cattle is common and has been reported in many studies [33,34,117,134]. We observed that all cases of co-infections including *Anaplasma* sp. with another pathogen such as piroplasms, *Borrelia* or even *Rickettsia*. Regarding the endemicity of VBDs, we observed the most infected region in Beni-Suef province, where the same genotypes or even new potential pathogens circulated between different animal hosts with a risk of transmission to other adjacent provinces and to humans. Furthermore, we observed that the highest prevalence among animal hosts was anaplasmoses (48/557; 8.6%), followed by piroplasmoses (23/557; 4.1%). Molecular analysis revealed an interesting diversity of these VB pathogens in ruminants and dogs. Therefore, further studies are needed for a better understanding of the epidemiological mapping of pathogen-host-vector in this region or even in the whole Egypt.

In conclusion, the current study is the first large-scale epidemiological observational study that performed molecular screening and characterization of multiple vector-borne pathogens in different animal hosts for better understanding of the endemicity of VBDs in Egypt. We identified for the first time *An*. *centrale*, *An*. *ovis*, a new *An*. *platys*-like and *Bo*. *theileri* in cattle, a new *An*. *platys*-like in buffaloes, *An*. *marginale*, *An*. *ovis*, a new *An*. *platys*-like and *Bo*. *theileri* in sheep, *An*. *platys*, a new *An*. *platys*-like and *S*. *digitata* in camels and *R*. *africae*-like, *An*. *platys*, *D*. *repens* and *Ac*. *reconditum* in dogs in Egypt. Therefore, ruminants and dogs in Egypt are reservoirs for multiple neglected, emerging and re-emerging vector-borne pathogens, especially new potential pathogens. Our observational study aimed to describe the repertory of possible vector-borne zoonotic pathogens in Egypt. However, convenient sampling approach did not permit us to evaluate the association of identified pathogens with host characteristics and to describe the geographic distribution of pathogens that limited our study. Further studies are needed to determine the pathogen-host-vector connections and other epidemiological factors of VBDs throughout Egypt, as well as to decipher the zoonotic potential of newly identified genotypes and their animals and public health significance.

## References

[1] Kuleš J, Potocnakova L, Bhide K, Tomassone L, Fuehrer HP, Horvatić A, et al. 2017. The Challenges and Advances in Diagnosis of Vector-Borne Diseases: Where Do We Stand? Vector Borne Zoonotic Dis. 2017; 17(5):285–296. doi: 10.1089/vbz.2016.2074 28346867

[2] WHO, The World Health Report-Changing History 95 World Health Organization. 2004; 96 p.

[3] Lew–Tabor AE, Rodriguez–Valle M. A review of reverse vaccinology approaches for the development of vaccines against ticks and tick-borne diseases. Ticks Tick-Borne Dis. 2016; 7:573–85. doi: 10.1016/j.ttbdis.2015.12.012 26723274

[4] Bergquist R, Stensgaard AS, Rinaldi L. Vector-borne diseases in a warmer world: Will they stay or will they go? Geospatial Health. 2018; 13(1):699. doi: 10.4081/gh.2018.699 29772870

[5] Harrus S, Baneth G. Drivers for the emergence and re-emergence of vector-borne protozoal and bacterial diseases. International Journal for Parasitology. 2005; 35: 1309–18. doi: 10.1016/j.ijpara.2005.06.005 16126213

[6] Baneth G, Bourdeau P, Bourdoiseau G, Bowman D, Breitschwerdt E, Capelli G, et al. Vector-Borne Diseases—constant challenge for practicing veterinarians: recommendations from the CVBD World Forum. Parasit Vectors. 2012; 5(55). doi: 10.1186/1756-3305-5-55 22433172 PMC3359183

[7] Schreeg ME, Marr HS, Tarigo JL, Cohn LA, Bird DM, Scholl EH, et al. Mitochondrial genome sequences and structures aid in the resolution of *Piroplasmida* phylogeny. PLoS One. 2016; 11:1–27. doi: 10.1371/journal.pone.0165702 27832128 PMC5104439

[8] Brown C. 2008. Tropical theileriosis. In: Brown C, Torres A. (Eds.), Foreign Animal Diseases, 7th ed. Boca Publications, Florida, USA. 2008; 401–4.

[9] Otranto D, Dantas-Torres F, Breitschwerdt EB. Managing canine vector-borne diseases of zoonotic concern: part two. Trends Parasitol. 2009; 25(5): 228–35. doi: 10.1016/j.pt.2009.02.005 19346164

[10] Bishop RP, Odongo DO, Mann DJ, Pearson TW, Sugimoto C, Haines LR, et al. *Theileria*. In: Nene V., Kole C. (Eds.), Genome Mapping and Genomics in Animal–Associated Microbes. Springer–Verlag Berlin Heidelberg, Berlin. 2009; 191–231.

[11] Antoniassi NAB, Correa AMR, da Silva Santos A, Pavarini SP, Sonne L, Bandarra PM, et al. Surto de babesiose cerebralem bovinos no Estado do Rio Grande do Sul. Ciencia Rural. 2009; 39:933–6.

[12] Schnittger L, Florin-Christensen M. Parasitic Protozoa of Farm Animals and Pets. 2018.

[13] Bono MF, Mangold AJ, Baravalle ME, Valentini BS, Thompson CS, Wilkowsky SE, et al. Efficiency of a recombinant MSA-2c-based ELISA to establish the persistence of antibodies in cattle vaccinated with *Babesia bovis*. Vet Parasitol. 2008; 157:203–10. doi: 10.1016/j.vetpar.2008.07.025 18783887

[14] OIE. Manual of Diagnostic Tests and Vaccines for Terrestrial Animals. Office International des Epizooties/World Organization for Animal Health, Paris. 2008.

[15] Ibrahim HM, Adjou Moumouni PF, Mohammed-Geba K, Sheir SK, Hashem ISY, Cao S, et al. Molecular and serological prevalence of *Babesia bigemina* and *Babesia bovis* in cattle and water buffalos under small-scale dairy farming in Beheira and Faiyum Provinces, Egypt. Veterinary Parasitology. 2013; 198(1–2): 187–92. doi: 10.1016/j.vetpar.2013.08.028 24075417

[16] El-Ashker M, Hotzel H, Gwida M, El-Beskawy M, Silaghi C, Tomaso H. Molecular biological identification of *Babesia*, *Theileria*, and *Anaplasma* species in cattle in Egypt using PCR assays, gene sequence analysis and a novel DNA microarray. Vet Parasitol. 2015; 207:329–34. doi: 10.1016/j.vetpar.2014.12.025 25591406

[17] Mahmoud MS, El-Ezz NT, Abdel-Shafy S, Nassar SA, El Namaky AH, Khalil WK, et al. Assessment of *Theileria equi* and *Babesia caballi* infections in equine populations in Egypt by molecular, serological and hematological approaches. Parasit Vectors. 2016; 9: 260. doi: 10.1186/s13071-016-1539-9 27146413 PMC4857240

[18] Abo El Fadl EA, El-Ashker M, Suganuma K, Kayano M. Discriminant analysis for the prediction and classification of tick-borne infections in some dairy cattle herds at Dakahlia Governorate, Egypt. Japanese Journal of Veterinary Research. 2017; 65(3): 127–33.

[19] Dumler JS, Barbet AF, Bekker CP, Dasch GA, Palmer GH, Ray SC, et al. Reorganization of genera in the families Rickettsiaceae and Anaplasmataceae in the order Rickettsiales: unification of some species of *Ehrlichia* with *Anaplasma*, *Cowdria* with *Ehrlichia* and *Ehrlichia* with *Neorickettsia*, descriptions of six new species combinations and designation of *Ehrlichia equi* and ‘HGE agent’ as subjective synonyms of *Ehrlichia phagocytophila*. Int J Syst Evol Microbiol. 2001; 51:2145–65. doi: 10.1099/00207713-51-6-2145 11760958

[20] Pruneau L, Moumène A, Meyer DF, Marcelino I, Lefrançois T, Vachiéry N. Understanding Anaplasmataceae pathogenesis using “Omics” approaches. Front Cell Infect Microbiol. 2014; 4:86. doi: 10.3389/fcimb.2014.00086 25072029 PMC4078744

[21] Aubry P, Geale DW. A review of bovine anaplasmosis. Transbound Emerg Dis. 2011; 58:1–30. doi: 10.1111/j.1865-1682.2010.01173.x 21040509

[22] de la Fuente J, Atkinson MW, Naranjo V, Fernández de Mera IG, Mangold AJ, Keating KA, et al. Sequence analysis of the Msp4 gene of *Anaplasma ovis* strains. Vet Microbiol. 2007; 119:375–81. doi: 10.1016/j.vetmic.2006.09.011 17052866

[23] Renneker S, Abdo J, Salih DE, Karagenç T, Bilgiç H, Torina A, et al. Can *Anaplasma ovis* in small ruminants be neglected any longer? Transbound Emerg Dis. 2013; 60(Suppl 2):105–12. doi: 10.1111/tbed.12149 24589109

[24] Dahmani M, Davoust B, Rousseau F, Raoult D, Fenollar F, Mediannikov O. Natural Anaplasmataceae infection in *Rhipicephalus bursa* ticks collected from sheep in the French Basque Country. Ticks Tick-borne Dis. 2017; 8:18–24. doi: 10.1016/j.ttbdis.2016.09.009 27666778

[25] Dahmani M, Davoust B, Sambou M, Bassene H, Scandola P, Ameur T, et al. Molecular investigation and phylogeny of species of the Anaplasmataceae infecting animals and ticks in Senegal. Parasit Vectors. 2019; 12:495. doi: 10.1186/s13071-019-3742-y 31640746 PMC6805679

[26] Rar V, Golovljova I. *Anaplasma*, *Ehrlichia*, and “Candidatus *Neoehrlichia*” bacteria: Pathogenicity, biodiversity, and molecular genetic characteristics, a review. Infect Genet Evol. 2011; 11:1842–61. doi: 10.1016/j.meegid.2011.09.019 21983560

[27] Constable PD, Hinchcli KW, Done SH, Grünberg W. Diseases of the Hemolymphatic and Immune Systems. In Veterinary Medicine, 11th ed.; Saunders, W.B., Ed.; Saunders Ltd.: London, UK, 2017.

[28] Radwan MEI, Ali A, Abd elhamied O. Epidemiological Studies, Molecular Diagnosis of *Anaplasma marginale* in Cattle and Biochemical Changes Associated with it in Kaliobia Governorate. Am J Infect Dis Microbiol. 2013; 1: 46–9.

[29] El-Naga TRA, Barghash SM. Blood Parasites in Camels (*Camelus dromedarius*) in Northern West Coast of Egypt. J Bacteriol Parasitol. 2016; 7:258.

[30] Elhariri MD, Elhelw RA, Hamza DA, Soliman DE. Molecular detection of *Anaplasma marginale* in the Egyptian water bufaloes (*Bubuloes bubalis*) based on major surface protein 1. J Egyp Soc Parasitol. 2017; 47:247–52.

[31] Fereig RM, Mohamed SGA, Mahmoud H, AbouLaila MR, Guswanto A, Nguyen TT, et al. Seroprevalence of *Babesia bovis*, *B*. *bigemina*, *Trypanosoma evansi*, and *Anaplasma marginale* antibodies in cattle in southern Egypt. Ticks Tick Borne Dis. 2017; 8:125–31. doi: 10.1016/j.ttbdis.2016.10.008 27789159

[32] Parvizi O, El-Adawy H, Melzer F, Roesler U, Neubauer H, Mertens-Scholz K. Seroprevalence and Molecular Detection of Bovine Anaplasmosis in Egypt. Pathogens. 2020; 9(1):64. doi: 10.3390/pathogens9010064 31963251 PMC7168636

[33] AL-Hosary A, Răileanu C, Tauchmann O, Fischer S, Nijhof MA, Silaghi C. Epidemiology and genotyping of *Anaplasma marginale* and co-infection with piroplasms and other Anaplasmataceae in cattle and buffaloes from Egypt. Parasit Vectors. 2020; 13:495. doi: 10.1186/s13071-020-04372-z 32993778 PMC7526245

[34] Tumwebaze AM, Lee SH, Adjou Moumouni PF, Mohammed-Geba K, Sheir SK, Galal-Khallaf A, et al. First detection of Anaplasma ovis in sheep and Anaplasma platys-like variants from cattle in Menoufia governorate, Egypt. Parasitol Int. 2020; 78:102150 doi: 10.1016/j.parint.2020.102150 32485226

[35] Parola P, Paddock DC, Socolovschi C, Labruna BM, Mediannikov O, Kernif T, et al. Update on Tick-Borne rickettsioses around the World: A Geographic Approach. Clin Microbiol Rev. 2013; 26:657–702. doi: 10.1128/CMR.00032-13 24092850 PMC3811236

[36] Abdel-Shafy S, Abdullah HAMH, El-Molla A, Salib AF, Ghazy AA. Epidemiology and diagnosis of rickettsiosis in animal hosts and tick vectors. Bulg J Vet Med. 2018; 22: 371–98.

[37] Merhej V, Raoult D. Rickettsial evolution in the light of comparative genomics. Biological Reviews of the Cambridge Philosophical Society. 2011; 86:379–405. doi: 10.1111/j.1469-185X.2010.00151.x 20716256

[38] Kelly PJ, Beati L, Mason PR, Matthewman LA, Roux V, Raoult D. *Rickettsia africae* sp. nov., the etiological agent of African tick bite fever. Int J Syst Bacteriol. 1996; 46(2): 611–4. doi: 10.1099/00207713-46-2-611 8934912

[39] Beati L, Meskini M, Thiers B, Raoult D. *Rickettsia aeschlimannii* sp. nov., a new spotted fever group rickettsia associated with *Hyalomma marginatum* ticks. International Journal of Systematic Bacteriology. 1997; 47:548–54. doi: 10.1099/00207713-47-2-548 9103647

[40] Raoult D, Roux V. Rickettsioses as paradigms of new or emerging infectious diseases. Clinical Microbiology Reviews. 1997; 10:694–719. doi: 10.1128/CMR.10.4.694 9336669 PMC172941

[41] Boudebouch N, Sarih M, Socolovschi C, Amarouch H, Hassar M, Raoult D, et al. Molecular survey for spotted fever group rickettsiae in ticks from Morocco. Clinical Microbiology and Infection. 2009; 15:259–60. doi: 10.1111/j.1469-0691.2008.02226.x 19456813

[42] Mediannikov O, Diatta G, Fenollar F, Sokhna C, Trape J-F, Raoult D. Tick-borne rickettsioses, neglected emerging diseases in rural Senegal. PLoS Negl Trop Dis. 2010; 4 (9):e821. doi: 10.1371/journal.pntd.0000821 20856858 PMC2939048

[43] Sambou M, Faye N, Bassène H, Diatta G, Raoult D, Mediannikov O. Identification of rickettsial pathogens in ixodid ticks in northern Senegal. Ticks Tick Borne Dis. 2014; 5(5):552–6. doi: 10.1016/j.ttbdis.2014.04.002 24908548

[44] Botros BA, Soliman AK, Darwish M, El Said S, Morrill JC, Ksiazek TG. Seroprevalence of murine typhus and fievre boutonneuse in certain human populations in Egypt. The Journal of tropical medicine and hygiene. 1989; 92:373–8. 2514278

[45] Soliman AK, Botros AB, Ksiazek GT. Seroprevalence of *Rickettsia typhi* and *Rickettsia conorii* infection among rodents and dogs in Egypt. The Journal of Tropical Medicine and Hygiene. 1989; 92:345–9. 2509729

[46] Corwin A, Habib M, Olson J, Scott D, Ksiazek T, Watts MD. The prevalence of arboviral, rickettsial, and Hantaanlike viral antibody among school children in the Nile river delta of Egypt. Transactions of the Royal Society of Tropical Medicine and Hygiene. 1992; 86:677–9. doi: 10.1016/0035-9203(92)90189-j 1363163

[47] Corwin A, Habib M, Watts D, Darwish M, Olson J, Botros B, et al. Community-based prevalence profile of arboviral, rickettsial, and Hantaan-like viral antibody in the Nile River Delta of Egypt. The American Journal of Tropical Medicine and Hygiene. 1993; 48:776–83. doi: 10.4269/ajtmh.1993.48.776 8101432

[48] Reynolds, M. G. Serologic evidence for exposure to spotted fever and typhus group rickettsioses among persons with acute febrile illness in Egypt. In: Proceedings of Fourth International Conference on Emerging Infectious Diseases, Atlanta. 2004.

[49] Lange JV, El Dessouky AG, Manor E. Spotted fever rickettsiae in ticks from the northern Sinai Governate, Egypt. Am J Trop Med Hyg. 1992; 46:546–51. doi: 10.4269/ajtmh.1992.46.546 1599048

[50] Loftis AD, Reeves WK, Szumlas DE. Surveillance of Egyptian fleas for agents of public health significance: *Anaplasma*, *Bartonella*, *Coxiella*, *Ehrlichia*, *Rickettsia*, and *Yersinia pestis*. Am J Trop Med Hyg. 2006; 75:41–8. 16837707

[51] Loftis AD, Reeves WK, Szumlas DE. Rickettsial agents in Egyptian ticks collected from domestic animals. Exp. Appl. Acarol. 2006; 40:67–81. doi: 10.1007/s10493-006-9025-2 17004028

[52] Socolovschi C, Barbarot S, Lefebvre M, Parola P, Raoult D. *Rickettsia sibirica mongolitimonae* in traveler from Egypt. Emer Infec Dis. 2010; 16:1495–6. doi: 10.3201/eid1609.100258 20735946 PMC3294977

[53] Abdel-Shafy S, Allam ATN, Mediannikov O, Parola P, Raoult D. Molecular detection of spotted fever group rickettsiae associated with ixodid ticks in Egypt. Vector-Borne and Zoonotic Diseases. 2012; 12:1–14. doi: 10.1089/vbz.2011.0705 22217182

[54] Abdullah AMH, El-Molla A, Salib AF, Allam ATN, Ghazy AA, Abdel-Shafy S. 2016. Morphological and molecular identification of the brown dog tick *Rhipicephalus sanguineus* and the camel tick *Hyalomma dromedarii* (Acari: Ixodidae) vectors of rickettsioses in Egypt. Vet World. 9: 1087–101. doi: 10.14202/vetworld.2016.1087-1101 27847418 PMC5104717

[55] Abdullah HHAM, El-Molla A, Salib AF, Allam ATN, Ghazy AA, Sanad MY, et al. Molecular Characterization of Rickettsiae Infecting Camels and Their Ticks Vectors in Egypt. Bacterial Empire. 2019; 2(1):10–8.

[56] Haitham E, Raoult D, Drancourt M. Relapsing fever borreliae in Africa. Am J Trop Med Hyg. 2013; 89:288–92. doi: 10.4269/ajtmh.12-0691 23926141 PMC3741250

[57] Trape JF, Diatta G, Arnathau C, Bitam I, Sarih M. The epidemiology and geo-graphic distribution of relapsing fever borreliosis in West and North Africa, with a review of the *Ornithodoros erraticus* complex (Acari: Ixodida). PLoS One. 2013; 8:e78473. doi: 10.1371/journal.pone.0078473 24223812 PMC3817255

[58] McCoy BN, Maïga O, Schwan TG. Detection of *Borrelia theileri* in *Rhipicephalus geigyi* from Mali. Ticks Tick Borne Dis. 2014; 5(4):401–3.Top of FormBottom of Form doi: 10.1016/j.ttbdis.2014.01.007 24709337 PMC4041617

[59] Ehounoud CB, Yao KP, Dahmani M, Achi YL, Amanzougaghene N, Kacou N’Douba A, et al. Multiple Pathogens Including Potential New Species in Tick Vectors in Côte d’Ivoire. PLoS Negl Trop Dis. 2016; 10(1):e0004367. doi: 10.1371/journal.pntd.0004367 26771308 PMC4714895

[60] Hagen RM, Frickmann H, Ehlers J, Krüger A, Margos G, Hizo-Teufel C, et al. Presence of *Borrelia* spp. DNA in ticks, but absence of *Borrelia spp*. and of *Leptospira spp*. DNA in blood of fever patients in Madagascar. Acta Trop. 2018; 177:127–34. doi: 10.1016/j.actatropica.2017.10.002 28986249

[61] Adham FK, Emtithal M, Abd-El-Samie EM, Refaat M, Gabre RM, El Hussein H. Detection of tick blood parasites in Egypt using PCR assay ii- *Borrelia burgdorfer isensu lato*. J. Egypt. Soc. Parasitol. 2010; 40(3):553–64. 21268526

[62] Hassan MI, Gabr HSM, Abdel-Shafy S, Hammad KM, Mokhtar MM. Prevalence of tick-vectors of *Theileria annulata* infesting the one-humped camels in Giza, Egypt. J. Egypt. Soc. Parasitol. 2017; 47(2):425–32.

[63] Mediannikov O, Fenollar F, Socolovschi C, Diatta G, Bassene H, Molez JF, et al. *Coxiella burnetii* in humans and ticks in rural Senegal. PLoS Negl. Trop. Dis. 2010; 4:e654. doi: 10.1371/journal.pntd.0000654 20386603 PMC2850317

[64] Eldin C, Mélenotte C, Mediannikov O, Ghigo E, Million M, Edouard S, Mege JL, Maurin M, Raoult D. 2017. From Q fever to *Coxiella burnetii* infection: A paradigm change. Clin Microbiol Rev, 30(1):115–90. doi: 10.1128/CMR.00045-16 27856520 PMC5217791

[65] Guatteo R, Seegers H, Taurel AF, Joly A, Beaudeau F. Prevalence of *Coxiella burnetii* infection in domestic ruminants: A critical review. Vet Microbiol. 2011; 149(1–2):1–16. doi: 10.1016/j.vetmic.2010.10.007 21115308

[66] Berri M, Arricau-Bouvery N, Rodolakis A. PCR-based detection of *Coxiella burnetii* from clinical samples. In: Sachse K. and Frey J., editors. Methods in Molecular Biology. Humana Press Inc., Totowa. 2003;153–61. 10.1385/1-59259-344-5:153 12512362

[67] Mazyad SA, Hafez AO. Q fever (*Coxiella burnetii*) among man and farm animals in North Sinai, Egypt. J Egypt Soc Parasitol. 2007; 37:135–42. 17580573

[68] Gwida M, El-Ashker M, El-Diasty M, Engelhardt C, Khan I, Neubauer H. Q fever in cattle in some Egyptian governorates: A preliminary study. BMC Res Notes. 2014; 7(12):881. doi: 10.1186/1756-0500-7-881 25481509 PMC4295271

[69] Horton KC, Wasfy M, Samaha H, Abdel-Rahman B, Safwat S, Fadeel MA, et al. Serosurvey for zoonotic viral and bacterial pathogens among slaughtered livestock in Egypt. Vector Borne Zoonotic Dis. 2014; 14(9):633–9. doi: 10.1089/vbz.2013.1525 25198525 PMC4676263

[70] Abdullah HHAM, Hussein AH, Abd El-Razik AK, Barakat MAA, Soliman AY. Q-Fever a neglected disease of Camels in Egypt. Vet World. 2019; 12(12):1945–50. doi: 10.14202/vetworld.2019.1945-1950 32095045 PMC6989333

[71] Abdel-Moein KA, Hamza DA. The burden of *Coxiella burnetii* among aborted dairy animals in Egypt and its public health implications. Acta Trop. 2017; 166(2):92–5. doi: 10.1016/j.actatropica.2016.11.011 27845064

[72] Abdullah HHAM, El-Shanawany EE, Abdel-Shafy S, Abou-Zeina HAA, Abdel-Rahman EH. Molecular and immunological characterization of *Hyalomma dromedarii* and *Hyalomma excavatum* (Acari: Ixodidae) vectors of Q fever in camels. Vet World. 2018; 11(8):1109–19. doi: 10.14202/vetworld.2018.1109-1119 30250371 PMC6141297

[73] Otranto D, Capelli G, Genchi C. Changing distribution patterns of canine vector borne diseases in Italy: leishmaniosis vs. dirofilariosis, Parasit. Vectors. 2009; 2(Suppl 1):S2. 10.1186/1756-3305-2-S1-S2 PMC2679394 19426441

[74] Perumal AN, Gunawardene YI, Dassanayake RS. *Setaria digitate* in advancing our knowledge of human lymphatic filariasis. J Helminthol. 2016; 90:129–38. doi: 10.1017/S0022149X15000309 25924635

[75] Brianti E, Gaglio G, Napoli E, Giannetto S, Dantas-Torres F, Bain O, et al. New insights into the ecology and biology of *Acanthocheilonema reconditum* (Grassi,1889) causing canine subcutaneous filariosis, Parasitology. 2012; 139:530–6. doi: 10.1017/S0031182011002198 22336052

[76] Tamilmahan P, Zama MMS, Pathak R, Muneeswaran NS, Karthik K. A retrospective study of ocular occurrences in domestic animals: 799 cases. Vet world. 2013; 6:274–6.

[77] Maharana BR, Potliya S, Ganguly A, Bisla SR, Mishra C, Ganguly I. First report of the isolation and phylogenetic characterization of equine *Setaria digitata* from India based on mitochondrial COI, 12S rDNA, and nuclear ITS2 sequence data. Parasitol Res. 2020; 119(2):473–81. doi: 10.1007/s00436-019-06587-1 31897790

[78] Albrechtová K, Sedlák K, PetrŽelková JK, Hlaváčb J, Mihalca DA, Lesingirian A, et al. Occurrence of filaria in domestic dogs of Samburu pastoralists in Northern Kenya and its associations with canine distemper. Vet Parasitol. 2011; 182:230–8. doi: 10.1016/j.vetpar.2011.05.042 21724332

[79] Siwila J, Mwase TE, Nejsum P, Simonsen EP. Filarial infections in domestic dogs in Lusaka, Zambia, Vet Parasitol. 2015; 210:250–4. doi: 10.1016/j.vetpar.2015.04.009 25944406

[80] Medkour H, Laidoudi Y, Athias E, Bouam A, Dizoe S, Davoust B, et al. Molecular and serological detection of animal and human vector-borne pathogens in the blood of dogs from Côte d’Ivoire. Comparative Immunology, Microbiology and Infectious Diseases. 2020; 69:101412. doi: 10.1016/j.cimid.2019.101412 31981798

[81] Thrusfield M, Christley R, Brown H, Diggle PJ, French N, Howe K, Kelly L, O’Connor A, Sargeant J, Wood H. Veterinary Epidemiology: Fourth Edition. (4th ed.) Wiley-Blackwell. 2017. doi: 10.1016/j.foodchem.2017.11.074

[82] Sokhna C, Mediannikov O, Fenollar F, Bassene H, Diatta G, Tall A, et al. Point-of-Care Laboratory of Pathogen Diagnosis in Rural Senegal. PLoS Negl Trop Dis. 2013; 7:e1999. doi: 10.1371/journal.pntd.0001999 23350001 PMC3547848

[83] Kumar S, Stecher G, Li M, Knyaz C, Tamura K. MEGA X: Molecular Evolutionary Genetics Analysis across computing platforms. Mol Biol Evol. 2018; 35:1547–9. doi: 10.1093/molbev/msy096 29722887 PMC5967553

[84] Tamura K, Nei M. Estimation of the number of nucleotide substitutions in the control region of mitochondrial DNA in humans and chimpanzees. Mol Biol Evol. 1993; 10:512–26. doi: 10.1093/oxfordjournals.molbev.a040023 8336541

[85] Ayalew W, Rege JEO, Getahun E, Tibbo M, Mamo Y. Delivering Systematic Information on Indigenous Animal Genetic Resources–the development and prospects of DAGRIS. Animal Genetic Resources Group, International Livestock Research Institute. P, O Box 5689. Addis Ababa, Ethiopia. 2003.

[86] Dantas-Torres F, Chomel BB, Otranto D. Ticks and tick-borne diseases: A One Health perspective. Trends Parasitol. 2012; 28(10):437–46. doi: 10.1016/j.pt.2012.07.003 22902521

[87] Elsify A, Sivakumar T, Nayel M, Salama A, Elkhtam A, Rizk M, et al. An epidemiological survey of bovine *Babesia* and *Theileria* parasites in cattle, buffaloes, and sheep in Egypt. Parasitology International. 2015; 64(1):79–85. doi: 10.1016/j.parint.2014.10.002 25305419

[88] Rizk MA, Salama A, El-Sayed SA, Elsify A, El-Ashkar M, Ibrahim H, et al. Animal level risk factors associated with Babesia and Theileria infections in cattle in Egypt. Acta Parasitologica. 2017; 62(4):796–804. doi: 10.1515/ap-2017-0096 29035848

[89] AL-Hosary A, Ahmed L, Ahmed J, Nijhof A, Clausen P. Epidemiological study on tropical theileriosis (*Theileria annulata* infection) in the Egyptian Oases with special reference to the molecular characterization of *Theileria* spp. Ticks and Tick-borne Diseases. 2018; 9(6):1489–93. doi: 10.1016/j.ttbdis.2018.07.008 30033328

[90] Mazyad SA, Khalaf SA. Studies on *Theileria* and *Babesia* infecting live and slaughtered animals in Al Arish and El Hasanah, North Sinai Governorate, Egypt. Journal of the Egyptian Society of Parasitology. 2002; 32(2):601–10. 12214937

[91] AL-Hosary A, Ahmed L, Seitzer U. First report of molecular identification and characterization of *Theileria* spp. from water buffaloes (*Bubalus bubalis*) in Egypt. Adv Anim Vet Sci. 2015; 3:629–33.

[92] Al-Hosary AA, El Sify A, Salama AA, Nayel M, Elkhtam A, Elmajdoub LO, Rizk MA, Hawash MM, Al-Wabel MA, Almuzaini AM, Ahmed LSE, Paramasivam A, Mickymaray S, Omar MA (2021) Phylogenetic study of Theileria ovis and Theileria lestoquardi in sheep from Egypt: Molecular evidence and genetic characterization. Vet World. 2021; 14: 634–9. doi: 10.14202/vetworld.2021.634-639 33935408 PMC8076446

[93] Passos LMF, Geiger SM, Ribeiro MFB, Pfister K, Zahler-Rinder M. First molecular detection of *Babesia vogeli* in dogs from Brazil. Veterinary Parasitology. 2005; 127(1):81–5. doi: 10.1016/j.vetpar.2004.07.028 15619377

[94] Salem NY, Farag HS. Clinico-pathological findings in *B*. *canis* infected dogs in Egypt. Comparative Clinical Pathology. 2014; 23(5):1305–7.

[95] Matjila PT, Penzhorn LB, Bekker PJC, Nijhof MA, Jongejan F. Confirmation of occurrence of *Babesia canis vogeli* in domestic dogs in South Africa. Vet Parasitol. 2004; 122:119–25. doi: 10.1016/j.vetpar.2004.03.019 15177716

[96] M’ghirbi Y, Bouattour A. Detection and molecular characterization of *Babesia canis vogeli* from naturally infected dogs and *Rhipicephalus sanguineus* ticks in Tunisia. Vet Parasitol. 2008; 152:1–7. doi: 10.1016/j.vetpar.2007.12.018 18242865

[97] Parola P, Raoult D. Ticks and tick-borne bacterial diseases in humans: an emerging infectious threat. Clin Infect Dis. 2001; 32(6):897–928. doi: 10.1086/319347 11247714

[98] CAPMAS. Animal Diseases. Available online: https://www.capmas.gov.eg/.

[99] Herndon DR, Palmer GH, Shkap V, Knowles DP, Brayton K. Complete genome sequence of *Anaplasma marginale* sub sp centrale. J Bacteriol. 2010; 192(1):379–80. doi: 10.1128/JB.01330-09 19854912 PMC2798241

[100] Bell-Sakyi L, Palomar AM, Bradford EL, Shkap V. Propagation of the Israeli vaccine strain of *Anaplasma centrale* in tick cell lines. Vet Microbiol. 2015; 179:270–6. doi: 10.1016/j.vetmic.2015.07.008 26210950 PMC4540598

[101] Belkahia H, Ben Said M, El Hamdi S, Yahiaoui M, Gharbi M, Daaloul-Jedidi M, et al. First molecular identification and genetic characterization of *Anaplasma ovis* in sheep from Tunisia. Small Rumin Res. 2014; 121:404–10.

[102] Aouadi A, Leulmi H, Boucheikhchoukh M, Benakhla A, Raoult D, Parola P. Molecular evidence of tick-borne hemoprotozoan-parasites (*Theileria ovis* and *Babesia ovis*) and bacteria in ticks and blood from small ruminants in Northern Algeria. Comparative Immunology, Microbiology and Infectious Diseases. 2017; 50:34–9. doi: 10.1016/j.cimid.2016.11.008 28131376

[103] Sadeddine R, Diarra AZ, Laroche M, Mediannikovc O, Righia S, Benakhla A, et al. Molecular identification of protozoal and bacterial organisms in domestic animals and their infesting ticks from north-eastern Algeria. Ticks and Tick-borne Diseases. 2020; 11(2):101330. doi: 10.1016/j.ttbdis.2019.101330 31786146

[104] Nair ADS, Cheng C, Ganta CK, Sanderson MW, Alleman AR, Munderloh UG, et al. Comparative Experimental Infection Study in Dogs with *Ehrlichia canis*, *E*. *chaffeensis*, *Anaplasma platys* and *A*. *phagocytophilum*. PLoS One. 2016; 11:e0148239. doi: 10.1371/journal.pone.0148239 26840398 PMC4739612

[105] Zobba R, Anfossi AG, Pinna Parpaglia ML, Dore GM, Chessa B, Spezzigu A, et al. Molecular investigation and phylogeny of *Anaplasma* spp in Mediterranean ruminants reveal the presence of neutrophil-tropic strains closely related to *A*. *platys*. Appl Environ Microbiol. 2014; 80:271–80. doi: 10.1128/AEM.03129-13 24162569 PMC3911010

[106] Dahmani M, Davoust B, Benterki MS, Fenollar F, Raoult D, Mediannikov O. Development of a new PCR-based assay to detect *Anaplasmataceae* and the first report of *Anaplasma phagocytophilum* and *Anaplasma platys* in cattle from Algeria. Comp Immunol Microbiol Infect Dis. 2015; 4:39–45. doi: 10.1016/j.cimid.2015.02.002 25748051

[107] Ben Said M, Belkahia H, El Mabrouk N, Saidani M, Alberti A, Zobba R, et al. *Anaplasma platys*-like strains in ruminants from Tunisia. Infect Genet Evol. 2017; 49:226–33. doi: 10.1016/j.meegid.2017.01.023 28130168

[108] Belkahia H, Ben Said M, Sayahi L, Alberti A, Messadi L. Detection of novel strains genetically related to *Anaplasma platys* in Tunisian one-humped camels (*Camelus dromedarius*). J Infect Dev Ctries. 2015; 9:1117–25. doi: 10.3855/jidc.6950 26517487

[109] Selmi R, Ben Said M, Dhibi M, Ben Yahia H, Messadi L. Improving specific detection and updating phylogenetic data related to *Anaplasma platys*-like strains infecting camels (*Camelus dromedarius*) and their ticks. Ticks Tick-borne Dis. 2019; 10:101260. doi: 10.1016/j.ttbdis.2019.07.004 31327747

[110] Berggoetz M, Schmid M, Ston D, Wyss V, Chevillon C, Pretorius AM, et al. Tick-borne pathogens in the blood of wild and domestic ungulates in South Africa: interplay of game and livestock. Ticks Tick-borne Dis. 2014; 5:166–75. doi: 10.1016/j.ttbdis.2013.10.007 24418761

[111] Socolovschi C, Huynh TP, Davoust B, Gomez J, RaoulT D, Parola P. Transovarial and trans-stadial transmission of *Rickettsiae africae* in *Amblyomma variegatum* ticks. Clin Microbiol Infect. 2009; 15 Suppl 2: 317–8. doi: 10.1111/j.1469-0691.2008.02278.x 19456811

[112] Kernif T, Djerbouh A, Mediannikov O, Ayach B, Rolain JM, Raoult D, et al. *Rickettsia africae* in *Hyalomma dromedarii* ticks from sub-Saharan Algeria. Ticks Tick-borne Dis. 2012; 3(5–6):377–9. doi: 10.1016/j.ttbdis.2012.10.013 23164496

[113] Ogo NI, de Mera IG, Galindo RC, Okubanjo OO, Inuwa HM, Agbede RI, et al. Molecular identification of tick-borne pathogens in Nigerian ticks. Vet Parasitol. 2012; 187(3–4):572–7. doi: 10.1016/j.vetpar.2012.01.029 22326937

[114] Smith RD, Miranpuri GS, Adams HJ, Ahrens HE. *Borrelia theileri*: isolation from ticks (*Boophilus microplus*) and tick-borne transmission between splenectomized calves, Am J Vet Res. 1995; 46(6):1396–8. 4026019

[115] Abdullah HHAM, Aboelsoued D, Farag KT, Abdel Megeed NK, Abdel-Shafy S, Parola P, et al. (2021): Molecular characterization of some equine vector-borne diseases and associated arthropods in Egypt. Preprint. doi: 10.21203/rs.3.rs-26089/v1 34954258

[116] Morel N, De Salvo MN, Cicuttin G, Rossner V, Thompson CS, Mangold AJ, et al. The presence of *Borrelia theileri* in Argentina. Vet Parasitol Reg Stud Reports. 2019; 17:100314. doi: 10.1016/j.vprsr.2019.100314 31303227

[117] Abanda B, Paguem A, Abdoulmoumini M, Kingsley TM, Renz A, Eisenbarth A. Molecular identification and prevalence of tick-borne pathogens in zebu and taurine cattle in North Cameroon. Parasit. Vectors. 2019; 12(1):448. doi: 10.1186/s13071-019-3699-x 31511038 PMC6737592

[118] Botros BA, Soliman AK, Salib AW, Olsen J, Hibbs RG, Williams JC, et al. *Coxiella burnetii* antibody prevalence in North-East Africa determined by enzyme immunoassay. J Trop Med Hyg. 1995; 98(3):173–8. 7783275

[119] Khalifa ON, Elhofy IF, Fahmy AH, Mona M, Sobhy MM, Agag MA. Seroprevalence and molecular detection of *Coxiella burnetii* infection in sheep, goats and human in Egypt. ISOI J Microbiol Biotechnol Food Sci. 2016; 2(1):1–7.

[120] Klemmer J, Njeru J, Emam A, El-Sayed A, Moawad AA, Henning K, et al. Q fever in Egypt: Epidemiological survey of *Coxiella burnetii* specific antibodies in cattle, buffaloes, sheep, goats and camels. PLoS One. 2018; 13(2):e0192188. doi: 10.1371/journal.pone.0192188 29466380 PMC5821454

[121] Mohammed OB, Jarelnabi AA, Aljumaah RS, Alshaikh MA, Bakhiet AO, Omer SA, et al. *Coxiella burnetii*, the causative agent of Q fever in Saudi Arabia: Molecular detection from camel and other domestic livestock. Asian Pac J Trop Med. 2014; 7(9):715–9.

[122] Hussein MF, Alshaikh MA, Al-Jumaah RS, GarelNabi A, Al-Khalifa I, Mohammed OB. The Arabian camel (*Camelus dromedarius*) as a major reservoir of Q fever in Saudi Arabia. Comp Clin Path. 2015; 24(4):887–92.

[123] Farkas R, Mag V, Gyurkovszky M, Takács N, Vörös K, Solymosi N. The current situation of canine dirofilariosis in Hungary. Parasitol Res. 2020; 119(1):129–35. doi: 10.1007/s00436-019-06478-5 31754854 PMC6942023

[124] Capelli G, Genchi C, Baneth G, Bourdeau P, Brianti E, Cardoso L, et al. Recent advances on *Dirofilaria repens* in dogs and humans in Europe, Parasit. Vectors. 2018; 11 (1):663. 10.1186/s13071-018-3205-x PMC6299983 30567586

[125] Magnis J, Lorentz S, Guardone L, Grimm F, Magi M, Naucke TJ, et al. Morphometric analyses of canine blood microfilariae isolated by the Knott’s test enables *Dirofilaria immitis* and *D*. *repens* species-specific and *Acanthocheilonem*a (syn. *Dipetalonema*) genus-specific diagnosis, Parasit. Vectors. 2013; 6(1):3–7. doi: 10.1186/1756-3305-6-48 PMC3598535 23442771

[126] Sivagurunathan A, Atwa MEA. A case of *Acanthocheilonema reconditum* in a dog. Int J Med Sci. 2017; 2(1):25–6.

[127] Schwan VE. Filariosis of Domestic Carnivores in Gauteng, KwaZulu-Natal and Mpumalanga Provinces, South Africa, and Maputo Province, Mozambique. 2009.

[128] BinoSundar ST, D’Souza PE. Morphological characterization of *Setaria* worms collected from cattle. J Parasit Dis. 2015; 39(3):572–6. doi: 10.1007/s12639-013-0399-x 26345074 PMC4554581

[129] Kaur D, Ganai A, Parveen S, Borkataki S, Yadav A, Katoch R, et al. *Setaria digitata* Occurrence of in a cow. J Parasit Dis. 2015; 39(3):477–8. doi: 10.1007/s12639-013-0376-4 26345055 PMC4554577

[130] El-Azazy OM, Ahmed YF. Patent infection with Setaria digitata in goats in Saudi Arabia. Vet Parasitol. 1999; 82(2):161–6. doi: 10.1016/s0304-4017(98)00264-7 10321587

[131] Wijesundera WS, Chandrasekharan NV, Karunanayake EH. A sensitive polymerase chain reaction-based assay for the detection *Setaria digitata*: of the causative organism of cerebrospinal nematodiasis in goats, sheep and horses. Vet Parasitol. 1999; 81:225–33. doi: 10.1016/s0304-4017(98)00248-9 10190866

[132] Radwan AM, Ahmed NE, Elakabawy LM, Ramadan MY, Elmadawy RS. Prevalence and pathogenesis of some filarial nematodes infecting donkeys in Egypt. Vet World. 2016; 9:888–92. doi: 10.14202/vetworld.2016.888-892 27651679 PMC5021840

[133] Shin J, Ahn KS, Suh GH, Kim HJ, Jeong HS, Kim S, et al. *Setaria digitata* First blindness cases of horses infected with (Nematoda: Filarioidea) in the Republic of Korea. Korean J Parasitol. 2017; 55(6):667–71. doi: 10.3347/kjp.2017.55.6.667 29320823 PMC5776899

[134] Bursakov SA, Kovalchuk SN. Co-infection with tick-borne disease agents in cattle in Russia. Ticks and Tick-borne Dis. 2019; 10(3):709–13. doi: 10.1016/j.ttbdis.2019.03.004 30878569

[135] Dahmana H, Amanzougaghene N, Davoust B, Carette O, Normand T, Demoncheaux JP, et al. Great diversity of Piroplasmida in Equidae in Africa and Europe, including potential new species. Vet. Parasitol.: Regional Studies and Reports. 2019; 18:100332. 10.1016/j.vprsr.2019.100332 31796173

[136] Rolain J-M, Stuhl L, Maurin M, Raoult D. Evaluation of antibiotic susceptibilities of three rickettsial species including *Rickettsia felis* by a quantitative PCR DNA assay. 2002. Antimicrob Agents Chemother. 46: 2747–51. doi: 10.1128/AAC.46.9.2747-2751.2002 12183224 PMC127393

[137] Roux V, Rydkina E, Eremeeva M, Raoult D. Citrate synthase gene comparison, a new tool for phylogenetic analysis, and its application for the rickettsiae. Int J Syst Bacteriol. 1997; 47:252–61. doi: 10.1099/00207713-47-2-252 9103608

[138] Roux V, Raoult D. Phylogenetic analysis of members of the genus Rickettsia using the gene encoding the outer-membrane protein *rOmpB* (*ompB*). Int J Syst Evol Microbiol. 2000; 50:1449–55. doi: 10.1099/00207713-50-4-1449 10939649

[139] Bottieau E, Verbruggen E, Aubry C, Socolovschi C, Vlieghe E. Meningoencephalitis complicating relapsing fever in traveler returning from Senegal. Emerg Infect Dis. 2012; 18(4):697–8. doi: 10.3201/eid1804.111771 22469185 PMC3309691

[140] Glazunova O, Roux V, Freylikman O, Sekeyova Z, Fournous G, Tyczka J, et al. *Coxiella burnetii* genotyping. Emerg Infect Dis. 2005; 11:1211–17. doi: 10.3201/eid1108.041354 16102309 PMC3320512

[141] Raoult D, Roblot F, Rolain JM, Besnier JM, Loulergue J, Bastides, et al. First isolation of *Bartonella alsatica* from a valve of a patient with endocarditis. J Clin Microbiol. 2006; 44(1):278–9. doi: 10.1128/JCM.44.1.278-279.2006 16390990 PMC1351971

[142] Laidoudi Y, Davoust B, Varloud M, Niang EHA, Fenollar F, Mediannikov O. Development of a multiplex qPCR-based approach for the diagnosis of *Dirofilaria immitis*, *D*. *repens* and *Acanthocheilonema reconditum*. Parasit. Vectors. 2020; 13(1):319. doi: 10.1186/s13071-020-04185-0 32571427 PMC7309989

[143] Laidoudi Y, Medkour H, Levasseur A, Davoust B, Mediannikov O. New Molecular Data on *Filaria* and its *Wolbachia* from Red Howler Monkeys (Alouatta macconnelli) in French Guiana-A Preliminary Study. Pathogens. 2020; 9,626. doi: 10.3390/pathogens9080626 32752052 PMC7460519

[144] Laidoudi Y, Ringot D, Watier-Grillot S, Davoust B, Mediannikov. A cardiac and subcutaneous canine dirofilariosis outbreak in a kennel in central France. Parasites. 2019; 26,72. 10.1051/parasite/2019073 PMC6913249 31840652

