# Supplementary material for: Multiple vector-borne pathogens of domestic animals in Egypt
Source: PLoS Negl Trop Dis. 2021 Sep 29;15(9):e0009767. doi: 10.1371/journal.pntd.0009767 (PMC8480906; doi:10.1371/journal.pntd.0009767)
Supplement: Attachment — Submitted filename: Revision Letter-HA.docx [file pntd.0009767.s001.docx]

Revision Letter

Dear Editor,

We are grateful for the valuable comments of our manuscript (PNTD-D-21-00168) entitled "Multiple vectors borne diseases in Domestic Animals in Egypt". We would like to inform you that reviewers’ comments have been made. The following are authors' responses point by point to all comments.

Regards,

Oleg MEDIANNIKOV

Reviewer #1: Abstract
Line 13: you can just say vector-borne pathogens.
Line 21: please replace ‘proved’ with found.
Line 26: the presence of different….
Line 28: ruminants seem to play….

Author’s response:

All requested corrections have been done

Author summary
Line 34: please remove ‘was’ after that.
Line 39: these findings suggest that….
Line 40: please rephrase the end part of the sentence to ‘emerging and re-emerging potentially new vector borne pathogens that have significant implications in human health’ or something along those lines.
Author’s response:

All requested corrections have been done

Introduction
Line 46: humans, livestock, companion animals and…
Line 46: vbds are a worldwide burden….
Line 50: such as.
Line 51: spread of VBDs…
Line 51: such as globalization…
Line 53: pose.

Author’s response:

All requested corrections have been done.

Line 54 – 112: these paragraphs can be deleted or significantly condensed.

Author’s response:

In these paragraphs, we have briefly given the information about each pathogen and the endemicity of these diseases in Egypt, because it is the first large-scale study covering multiple VBDs. However, we have condensed them as much as possible.

Line 118: this sentence needs to be rephrased.
Line 119: provide.

Author’s response:

All requested corrections have been done.

Materials and Methods
This section is fairly straightforward and concise but the other sections are heavily wordy and can be greatly condensed.
Line 128: I’m assuming all these animals were domesticated? Please mention it explicitly.
Line 161: ‘performed’ instead of applied.

Author’s response:

All requested corrections have been done.

Reviewer #2: The objectives of the study are articulated with the hypothesis.

It is not clear what are the criteria for inclusion of animals in the research and how the sample size calculation was performed to obtain the prevalence.

Author’s response:

We mentioned in the materials and methods section that a convenience sampling strategy was used for finding out the prevalence of VBDs at the time of the study through a cross-sectional sampling from animals’ population.

In addition, they mention that information such as gender, breed, age, health status and vector infestation were collected, but it is not described or analyzed in the manuscript.

Author’s response:

That is true, we collected information on the animals studied, but our study has mainly focused on the screening and characterization of VBDs. We also discussed the prevalence of the infection in different localities in Egypt.

Therefore, the analysis of epidemiological factors will be investigated in further studies for each VBD.

There are no concerns about complying with ethical or regulatory requirements.

Reviewer #3: The objectives of the study were clearly stated.
The study design did not appropriately address the stated objective
The population were not described and hence cannot be measured with the study hypothesis
The sample size is not sufficient in ensuring adequate power to address the aim off the study
More analysis needs to be carried out. Authors should mention exactly where sampling was done in the provinces whether households, kraals, abattoir and their respective confounders to the study.

Author’s response:

We thank the Reviewer for the commentaries. In fact, our aim was to describe and characterize the spectrum of zoonotic pathogen (we have changed the objectives in the Abstract), because this domain of infectious diseases is still very few explored. We think that now these objectives correspond to a study.

Respectively, the populational characteristic may not play important role in the “explorative study” like this. However, now, when we know exactly the spectrum of different pathogens implicated in animal host health, more precise studies using the populational characteristics may be performed.

We added more details about the locations of sampling (table 1).


**Results**


-Does the analysis presented match the analysis plan?

-Are the results clearly and completely presented?

-Are the figures (Tables, Images) of sufficient quality for clarity?


Reviewer #1: I think the whole results section needs to be reworked. As it is written, it is very difficult to read and grasp the main points of the research. The emphasis is heavily placed on the pathogens and not on the animals, but I think that it should be the other way around (this is not a microbiology journal). For example, instead of saying “No Bartonella sp. DNA was detected in all animal hosts, while, piroplasms, Anaplasma sp., Rickettsia sp., Borrelia sp., C. burnetii and Filaria sp. DNA were detected in different animal hosts (Table 3)” please write it as ‘None of the animals were positive for Bartonella sp. while different animal species were positive for……..’ or something along those lines.

Author’s response:

We thank the Reviewer for this valuable remark.

We tried to rework the manuscript focusing on the animal hosts all over the manuscript.

The whole result section was edited to be clearer and focus on animal hosts perspective.

In line 133: You mention that you also recorded data about each animal’s gender, breed, age, vector infestation and health status but you do not report whether any of these factors had an effect on the detection of pathogens.
Author’s response:

That is true, we collected information on the animals studied, but as we already mentinned before, our study has mainly focused on the screening and characterization of VBDs. Our samples were too heterogenous, so comparative analysis may not be done, so we concentrated on the descriptive explorative study. We also discussed the prevalence of the infection in different localities in Egypt.

Therefore, the analysis of epidemiological factors will be investigated in further studies for each VBD.

Reviewer #2: The results of the analysis are adequate and clearly described. The figures and tables were well chosen and have quality. I suggest the inclusion of a map of Egypt showing the origin of the animals.

Author’s response:

Map of Egypt was added

Reviewer #3: The analysis matches the analysis plan, but more analysis needs to be done.
The results are clearly presented but not complete.
The figures (Tables, Images) are of sufficient quality for clarity
Author’s response:

the analysis of epidemiological factors will be investigated in further studies for each VBD. However, we discussed the prevalence of the infection in different localities in Egypt.

--------------------

**Conclusions**


-Are the conclusions supported by the data presented?

-Are the limitations of analysis clearly described?

-Do the authors discuss how these data can be helpful to advance our understanding of the topic under study?

-Is public health relevance addressed?


Reviewer #1: Needs to be rewritten. I could not make sense of the results section. Therefore, I don’t think any of my specific comments on the discussion/conclusions section would be helpful. I would request the authors to please rewrite the results and discussion sections from mainly the host animals’ perspective and not from the pathogens’ perspective. Also, please try to be concise as the way it is written is very wordy.
Author’s response:

The whole result and discussion section were edited to be clearer and supply the animals’ perspective than pathogens.

I would like to see table 3 with the rows and columns switched.

Author’s response:

All requested corrections have been done.

Reviewer #2: The conclusion is adequate, but the authors do not describe the limitations of the analysis. The relevance of the results for advancing the discussion of the subject and the importance of the findings for public health are highlighted.

Reviewer #3: The conclusion is not supported by the data presented.

The limitations of the study were not mentioned

Author’s response:

We thank the reviewers 2 and 3 for the commentaries. We included the phrases concerning the limitations of our study in the text as follows:

Lines 590-525 (track-changes manuscript)

“Our observational study aimed to describe the repertory of possible vector-borne zoonotic pathogens in Egypt. However, convenient sampling approach did not permit us to evaluate the association of identified pathogens with host characteristics and to describe the geographic distribution of pathogens that limited our study.”

--------------------

**Editorial and Data Presentation Modifications?**


Use this section for editorial suggestions as well as relatively minor modifications of existing data that would enhance clarity. If the only modifications needed are minor and/or editorial, you may wish to recommend “Minor Revision” or “Accept”.

Reviewer #1: This manuscript can be greatly condensed to better explain the results and their importance in the discussion sections.

Author’s response:

The whole result and discussion sections were edited to be clearer.

Reviewer #2: Line 21: replace “Dirofilariarepens” by “Dirofilaria repens”

Author’s response:

All requested corrections have been done.

Line 55: replace “Piroplamida” by “Piroplasmida”

Author’s response:

All requested corrections have been done.

Lines 119-121: It is not the first data... There are at least two published works with objectives similar to the present manuscript:
Tumwebaze MA, et al. Parasitol Int. 2020 Oct;78:102150. doi: 10.1016/j.parint.2020.102150
AL‑Hosary et al. Parasites Vectors (2020) 13:495 <https://doi.org/10.1186/s13071-020-04372-z>

Author’s response:

We thank the Reviewer for this suggestion and added these references in the manuscript. However, both articles only concerned one disease (Anaplasmosis or Piroplasmosis), but our study covered **multiple** vector-borne diseases in different animal hosts.

Lines 128-131: It is not clear what are the criteria for inclusion of animals in the research and how the sample size calculation was performed to obtain the prevalence. I suggest the inclusion of a map of Egypt showing the origin of the animals.

Author’s response:

We added more details about the locations of sampling (table 1). Sampling was performed by convenient strategy that limited out study We mentioned these limitations in conclusions and described the strategy in the manuscript

Egyptian map was added (Figure1).

Lines 132-133: The authors mention that information such as gender, breed, age, health status and vector infestation were collected, but these data are not described or analyzed in the manuscript.

Author’s response:

That is true, we collected information on the animals studied, but our study has mainly focused on the screening and characterization of VBDs. We also discussed the prevalence of the infection in different localities in Egypt.

Therefore, the analysis of epidemiological factors will be investigated in further studies for each VBD.

Lines 135-136: Why was the blood extraction kit not used?

Author’s response:

We used EZ blood extraction kit. It was corrected in the manuscript.

Lines 149-151, and line 166: Was a reagent blanck control used?
Line 190: Was a reagent blanck control used?

Author’s response:

We used negative control in each reaction that contains all the reagent without DNA template.

Line 191: How was the sample size determined for the calculation of prevalence? What is the justification for using the term "Prevalence" throughout the text?

Author’s response:

We have collected all animals in the areas where we performed the study and when the owners gave their consent. We did not calculate the sample size and the prevalence in our manuscript is the percentage of the positive samples in total sampled specimens.

Lines 285-290: It is important to describe the presence of co-infections also in the abstract

Author’s response:

The data was added in the abstract.

Lines 318-319: Replace the term “E. canis” by “Ba. canis”. In addition, citation number 89 refers to the first molecular description of Babesia vogeli in Brazil, so it does not seem appropriate to use this reference here.

Author’s response:

It is corrected to *Ba. canis*.

Regards to reference 89, the title of paper is the first molecular description of Babesia vogeli in Brazil, but the accession no (GenBank: AY371197) was cited in this paper referred to that genotype derived from Egyptian dog.

Lines 338-339, 356-357, 427-431: This study reports the first detection of A. ovis in sheep and A. platys-like strains in cattle in Menoufia and Egypt:
Tumwebaze MA, Lee SH, Adjou Moumouni PF, Mohammed-Geba K, Sheir SK, Galal-Khallaf A, Abd El Latif HM, Morsi DS, Bishr NM, Galon EM, Byamukama B, Liu M, Li J, Li Y, Ji S, Ringo AE, Rizk MA, Suzuki H, Ibrahim HM, Xuan X. First detection of Anaplasma ovis in sheep and Anaplasma platys-like variants from cattle in Menoufia governorate, Egypt. Parasitol Int. 2020 Oct;78:102150. doi: 10.1016/j.parint.2020.102150

Author’s response:

This reference has recently been published after I finished from writing of manuscript. Also, our accession numbers (MN626399-MN626400-MN626392) were released before their accession numbers. However, we added in our manuscript as a parallel study.

Lines 383-384: Borrelia theileri description in Brazil was from ticks, not from bovine samples

Author’s response:

All requested corrections have been done.

Line 397: Replace “faces” by “feces”

Author’s response:

All requested corrections have been done.

Lines 425-433: It is importante to describe the limitations of the analysis in conclusion.
Author’s response:

We have added the limitations of our study in the conclusion section (see above).

Reviewer #3: (No Response)

--------------------

**Summary and General Comments**


Use this section to provide overall comments, discuss strengths/weaknesses of the study, novelty, significance, general execution and scholarship. You may also include additional comments for the author, including concerns about dual publication, research ethics, or publication ethics. If requesting major revision, please articulate the new experiments that are needed.

Reviewer #1: Overall, I think that the manuscript needs to be improved a lot more to be considered for reevaluation.

Author’s response:

The whole manuscript was edited to be easier to read.

Reviewer #2: The present work demonstrates important data about agents transmitted by vectors in animals in Egypt, contributing to reduce an existing gap on this knowledge.
There are some recent works describing some pathogens that the authors thought were unpublished data. However, this does not detract from the research that was really extensive, covering various genera of pathogens transmitted by vectors to farm and companion animals.

Reviewer #3: TITLE
‘Multiple vectors borne diseases in domestic animals in Egypt’... Title is misleading it was not the disease that was investigated but the presence of pathogens.

Author’s response:

All requested corrections have been done.

The title now is as follows: “Multiple Vectors-borne Pathogens in Domestic Animals in Egypt”

ABSTRACT
Abstract needs to be re-written. Certain sentences do not read well and need restructuring. Authors should check how capitals and small letters are used and rectify all anomalies. The type of study should be stated in the abstract. Line 26 – 27 states that ‘For the first time, we detected the presence different zoonotic, mostly vector-borne pathogens in the blood of domestic animals in Egypt…. The question is by the use of mostly is the author implying that some of the pathogens were not VBD? If this is so which ones are those and why include them in the write up since the aim of the study was on VBD. All the new genotype discovered during the study should be mentioned under the abstract section.

Author’s response:

The abstract was edited.

INTRODUCTION
Some of the diseases mentioned such as piroplasmosis presents itself as asymptomatic in livestock, some pathogens like Theileria present itself in the asymptomatic and is not zoonotic therefore what is its economic importance to merit it being studied. In inference the mortality and morbidity of these diseases should be highlighted clearly so that the relevance of its pathogens being studied is put in proper perspective.

Author’s response:

We mentioned in the introduction section the economic important of piroplasmoses as it cause high mortality in animal herds and cause severe anemia in acute cases.

On line 46 what is the meaning of companion in that context.

Author’s response:
Companion animals are those animals who share our homes and lives as dogs and cats, and may also include birds and horses.

METHODS
The section was not well written. Authors should rewrite it by arranging it systematically following the order of; study area or study setting description-make sure each province is discussed in detail including their population, seasons and topography etc, sampling design and procedure-should be as detailed as possible including how each animal was sampled per province, where e etc, exclusion criteria, statistical analysis, ethical approval and consent.

Author’s response:

Table 1 was edited and figure 1 was added.

The convenient sampling that was carried out does not allow for authors to generalize the study to the whole country. The authors should be clear on the distribution of the animals in the provinces in the country and why.

Authors should stop beginning sentences with acronym.

Author’s response:

All requested corrections have been done.

Describe how field and laboratory processes were carried out.

OVERALL
For substantial portion of the article it is not sequentially written out to allow for flow of thought.
Author’s response:

The whole manuscript was edited to be clearer.

RESULTS
There should be another table that shows the demographics, vector infestation and health status of the animals with respective analysis.

Author’s response:

Table 1 and figure 1 were added to describe more details about samples collection locations.

Reconcile from line 187 to that of the breakdown of the various VBD pathogens mentioned at the abstract section as well as with the various tables.

Author’s response:

The whole manuscript was edited to be clearer.

DISCUSSION
Line 312 and 319 should state the authors whose studies this research is referring to before the references are written in parenthesis.

Author’s response:

All requested corrections have been done.

Check tenses, missing words and misuse of words and correct them.

Author’s response:

The whole manuscript was edited to be clearer.

Authors should also include in the discussion dynamics of all the VBD as present in the provinces, as per their proximity to each other and the potential beneficial or detrimental effect they might pose on the people of living in and around the study provinces and then the nation as a whole.

Author’s response:

The discussion was edited.

It should also be discussed the new strain found their potential virulence or pathogenecity.
How all pathogens identified economically impact the general populace should be further discussed.
Author’s response:

The discussion was edited.

CONCLUSION
Based on the sampling technique the conclusion is farfetched, the convenient sampling done in this study cannot be used in projecting what pertains in the whole country

Author’s response:

We added the description of these limitation in the conclusion section of our manuscript.

--------------------

PLOS authors have the option to publish the peer review history of their article ([what does this mean?](https://journals.plos.org/plosntds/s/editorial-and-peer-review-process#loc-peer-review-history)). If published, this will include your full peer review and any attached files.


If you choose “no”, your identity will remain anonymous but your review may still be made public.


**Do you want your identity to be public for this peer review?** For information about this choice, including consent withdrawal, please see our [Privacy Policy](https://www.plos.org/privacy-policy).

Reviewer #1: No

Reviewer #2: No

Reviewer #3: No


Figure Files:
While revising your submission, please upload your figure files to the Preflight Analysis and Conversion Engine (PACE) digital diagnostic tool, https://pacev2.apexcovantage.com. PACE helps ensure that figures meet PLOS requirements. To use PACE, you must first register as a user. Then, login and navigate to the UPLOAD tab, where you will find detailed instructions on how to use the tool. If you encounter any issues or have any questions when using PACE, please email us at [figures@plos.org](mailto:figures@plos.org).

Author’s response:

All requested corrections have been done.
 
Data Requirements:
Please note that, as a condition of publication, PLOS' data policy requires that you make available all data used to draw the conclusions outlined in your manuscript. Data must be deposited in an appropriate repository, included within the body of the manuscript, or uploaded as supporting information. This includes all numerical values that were used to generate graphs, histograms etc.. For an example see here: <http://www.plosbiology.org/article/info%3Adoi%2F10.1371%2Fjournal.pbio.1001908#s5>.

Author response:

All data was deposited within the manuscript.
 
Reproducibility:
To enhance the reproducibility of your results, PLOS recommends that you deposit laboratory protocols in protocols.io, where a protocol can be assigned its own identifier (DOI) such that it can be cited independently in the future. For instructions see https://journals.plos.org/plosntds/s/submission-guidelines#loc-methods
