# Supplementary material for: Multiple vector-borne pathogens of domestic animals in Egypt
Source: PLoS Negl Trop Dis. 2021 Sep 29;15(9):e0009767. doi: 10.1371/journal.pntd.0009767 (PMC8480906; doi:10.1371/journal.pntd.0009767)
Supplement: Attachment — Submitted filename: Revision letter-HA-OM.docx [file pntd.0009767.s002.docx]

Revision Letter

Dear Editor,

We are grateful for the valuable comments of our manuscript (PNTD-D-21-00168R1) entitled "Multiple Vector-borne Pathogens of Domestic Animals in Egypt". We would like to inform you that reviewers’ comments have been made, and English has been re-reviewed. The following are authors' responses point by point to all comments.

Regards,

Oleg MEDIANNIKOV

Reviewer's Responses to Questions

**Key Review Criteria Required for Acceptance?**


As you describe the new analyses required for acceptance, please consider the following:

**Methods**
-Are the objectives of the study clearly articulated with a clear testable hypothesis stated?

-Is the study design appropriate to address the stated objectives?

-Is the population clearly described and appropriate for the hypothesis being tested?

-Is the sample size sufficient to ensure adequate power to address the hypothesis being tested?

-Were correct statistical analysis used to support conclusions?

-Are there concerns about ethical or regulatory requirements being met?


Reviewer #2: The objectives of the study are related to the hypothesis. The authors clarified the criteria for inclusion of animals (convenience sampling) in the research, how the prevalence was calculated and added, as suggested, a figure containing the location of the samples obtained on the map of the country.
The authors, when asked about not presenting results from data mentioned in the material and methods (about sex, race, age, health status and vector infestation), justified that these data will be presented on another article. Therefore, I suggest that this part is not mentioned as part of this manuscript.
There are no concerns about complying with ethical or regulatory requirements.

Author’s response:

We thank the reviewer for this suggestion, therefore the line 136 has been removed.

Reviewer #3: Authors clearly articulated the objectives of the study with the right study design and sufficient sample size but the animal population was not clearly described with respect to their sex, breed, age, vector infestation and health as stated in line 135.

Author’s response:

We collected information on the animals studied, but the data and analysis of these epidemiological factors will be investigated in future studies for each VBD. Therefore, the line 136 has been removed.

--------------------

**Results**


-Does the analysis presented match the analysis plan?

-Are the results clearly and completely presented?

-Are the figures (Tables, Images) of sufficient quality for clarity?


Reviewer #2: Data results are adequate and clearly described. The figures and tables were well chosen and of good quality. The authors accepted the suggestion to include a map of Egypt with the location of the samples collected.

Reviewer #3: The analysis presented does match the analysis plan with the figures (Tables, Images) being of sufficient quality for clarity however authors on several occasions at the results section restated their methods adding up to the bulkiness of the manuscript.

Author’s response:

We tried to clarify the result section and remove the bulkiness sentences.

In line 209 how was 172 out of 557 arrived at for Anaplasmataceae in the animal hosts?

Author’s response:

We obtained this result by qPCR due to the higher sensitivity of it compared to standard PCR. We mentioned this details in the discussion section (line 325-328).

The table 3 shows An. platys in 7 dogs why is line 226 reporting 6?

Author’s response:

We mentioned that two different genotypes of *An. platys* were detected, one from 6 dogs and another from one dog, so total 7 dogs were positive for *An. Platys* (line 229-233).

Reconcile line 230 and 231 to table 3.

Author’s response:

We revised the text with table 3 and they are matched together.

Include the An. platys-like pathogen obtained from two dogs in table 3.

Author’s response:

We didn’t mention or detect *An. platys*-like pathogen in dogs in table 3. We detected only two different genotypes of *An. platys in seven dogs.*
--------------------

**Conclusions**


-Are the conclusions supported by the data presented?

-Are the limitations of analysis clearly described?

-Do the authors discuss how these data can be helpful to advance our understanding of the topic under study?

-Is public health relevance addressed?


Reviewer #2: The conclusion is adequate and the authors pointed out the limitations of the analysis. The relevance of the results for advancing the discussion of the subject and the importance of the findings for public health are highlighted.

Reviewer #3: yes the conclusions are supported by the data presented with limitations adequately described

--------------------

**Editorial and Data Presentation Modifications?**


Use this section for editorial suggestions as well as relatively minor modifications of existing data that would enhance clarity. If the only modifications needed are minor and/or editorial, you may wish to recommend “Minor Revision” or “Accept”.

Reviewer #2: The required changes were made, with no further changes suggested by my review.

Reviewer #3: Minor edits
Reword line 67, and lines 69 to 70 to convey appropriately the message it wishes to convey.

Author’s response:

All requested corrections have been done.

Authors should check spacing and punctuations throughout the manuscript.

Author’s response:

Spacing and punctuations have been corrected throughout the manuscript.

Abbreviations of all scientific names should be synchronized throughout the manuscript eg A. ovis and An. ovis, and A. platy and An. platy.

Author’s response:

The abbreviations of all scientific names have been corrected throughout the manuscript.

The prevalence of piroplasmoses have been stated on line 193 and 194, it needn't be repeated on line 197.

Author’s response:

The repeated data has been removed.

Sentence on lines 214 to 216 should be rewritten to convey the information it is intended to send to readers.
On line 424 replace ...' and there is'... with ...' with'...

Author’s response:

All requested corrections have been done.

--------------------

**Summary and General Comments**


Use this section to provide overall comments, discuss strengths/weaknesses of the study, novelty, significance, general execution and scholarship. You may also include additional comments for the author, including concerns about dual publication, research ethics, or publication ethics. If requesting major revision, please articulate the new experiments that are needed.

Reviewer #2: (No Response)

Reviewer #3: Authors should not repeat most of the results in the discussion section, just discuss.

Author’s response:

The repeated data and results in discussion section have been removed.

Discussion on piroplasmoses with respect to T. ovis should be more detailed.

Author’s response:

More details have been added (line 324-326).

Are all the organisms transmitted by ticks? If not kindly mention the other vectors and discuss them with their corresponding pathogens.

Author’s response:

More details have been added to manuscript.

Again all zoonotic pathogens should be mentioned as well as their devastating effect.
Author’s response:

More details have been added to manuscript.
--------------------
